# Silencing of *circRACGAP1* sensitizes gastric cancer cells to apatinib via modulating autophagy by targeting *miR-3657* and *ATG7*

**DOI:** 10.1038/s41419-020-2352-0

**Published:** 2020-03-05

**Authors:** Ling Ma, Zhangding Wang, Mengyan Xie, Yunlin Quan, Weiyou Zhu, Fengming Yang, Chenhui Zhao, Yu Fan, Na Fang, Huning Jiang, Qiang Wang, Shouyu Wang, Jianwei Zhou, Xiaofeng Chen, Yongqian Shu

**Affiliations:** 1 Department of Oncology, The First Affiliated Hospital of Nanjing Medical University, Nanjing, 210029 China; 2 Department of Gastroenterology, The Affiliated Drum Tower Hospital of Nanjing University Medical School, Nanjing, Jiangsu Province China; 3 Department of Molecular Cell Biology and Toxicology, Cancer Center, School of Public Health, Nanjing Medical University, Nanjing, 211166 China; 4 School of Mechanical Engineering, Jiangsu Key Laboratory for Design and Manufacture of Micro-Nano Biomedical Instruments, Southeast University, Nanjing, 211189 China; 5 Cancer Institute, The Affiliated People’s Hospital of Jiangsu University, Zhenjiang, 212002 China; 6 Department of Oncology, Pukou Branch Hospital of Jiangsu Province Hospital (Nanjing Pukou Central Hospital), Nanjing, 211800 China; 7 Jiangsu Key Lab of Cancer Biomarkers, Prevention and Treatment, Collaborative Innovation Center for Cancer Personalized Medicine, Nanjing Medical University, Nanjing, 211166 China

**Keywords:** Targeted therapies, Gastrointestinal cancer

## Abstract

The positive results of the apatinib phase III trial have cast new light on treatment for patients with advanced gastric cancer (GC). However, in terms of safety, apatinib toxicities may lead to a dose modification or treatment interruption. Therefore, proper intervention is urgently needed to help patients benefit from apatinib treatment. In this study, we found that apatinib promoted autophagy activation via upregulation of ATG7 expression and autophagy inhibition enhanced apatinib-induced apoptosis. With microRNA and circular RNA-sequencing analyses of GC xenograft models, we demonstrated that *circRACGAP1* functioned as an endogenous sponge for *miR-3657* to inhibit its activity and further upregulate *ATG7* expression. Silencing of *circRACGAP1* inhibited apatinib-induced autophagy, which was rescued by *miR-3657*. Moreover, knockdown of *circRACGAP1* sensitized GC cells to apatinib via autophagy inhibition in vitro and in vivo. These findings provided the first evidence that the *circRACGAP1*-*miR-3657-ATG7* axis mediates a novel regulatory pathway critical for the regulation of apatinib sensitivity in GC. Thus, specific blockage of *circRACGAP1* may be a potential therapeutic strategy to reduce the toxicities of apatinib and enhance its therapeutic effect in human GC.

## Introduction

Gastric cancer (GC) is the fifth most common cancer and the third leading cause of cancer-related deaths worldwide^1^. The morbidity of GC have already risen to the second place in China^2^. Despite certain advances in chemotherapy regimens and targeted therapies^3^, the 5-year survival of patients with advanced GC is disappointing^4^. Thus, further studies are needed to identify predictive biomarkers for selecting appropriate and effective treatments for patients with advanced GC.

Up to now, the results of most phase III studies of targeted therapies for advanced GC have been unsatisfactory, except for ToGA^5^, RAINBOW^6^, REGARD^7^, and the phase III trial of apatinib^8^. Apatinib is a novel and highly selectively inhibitor of the vascular endothelial growth factor receptor-2 (VEGFR-2) tyrosine kinase^9^ and has shown antitumor effects in various tumors^10,11^. Li et al.^8^ reported that apatinib treatment significantly improved overall survival and progression-free survival in patients with advanced GC refractory to two or more lines of previous systemic chemotherapies. However, a dose reduction is required to prevent apatinib toxicity such as grade 3–4 hand–foot skin reaction, grade 3–4 hypertension, and proteinuria (8.5%, 4.5%, and 2.3%, respectively)^8,12^. Therefore, certain measures, such as co-treatment, should be taken to reduce toxicity and help patients benefit from apatinib treatment.

The relationship between autophagy and apoptosis is complex. Autophagy can either promote or inhibit apoptosis under certain circumstances^13^, the mechanisms of which are related to the degradation of different pro-apoptotic or anti-apoptotic regulators by autophagy^14,15^. The role of autophagy in protecting cells from undergoing programmed cell death explains why the inhibition of autophagy has been shown to improve the response to other agents in some clinical trials^16,17^. In addition, concurrent autophagy inhibition has been reported to overcome the resistance of some tyrosine kinase inhibitors in human lung cancer, thyroid cancer, and bladder cancer^18–20^. Thus, our study was aimed at the potential role of autophagy inhibition in the co-treatment with apatinib.

Circular RNAs (circRNAs) were first discovered nearly 40 years ago^21^. Recently, with the development of deep RNA-sequencing technology, a growing number of studies have demonstrated that circRNAs are involved in biological processes and disease^22,23^. CircRNAs are characterized by a covalently closed loop structure with the 3′-end of the RNA joined to the 5′-end^24^. CircRNAs are more stable than their linear counterparts and the expression of circRNAs does not often correlate well with the expression of linear forms of the host genes^25^. Some studies have reported that circRNAs regulate cell growth, migration, invasion, and tumorigenesis^22,23,26^. In addition, circRNAs play significant role in autophagy^27,28^. Most of the circRNAs reported so far contain exons and can act as microRNA (miRNA) sponges to regulate gene expression^29,30^. However, the characterization and function of circRNAs in human cancer remain largely unknown.

In this study, we demonstrated for the first time that apatinib promoted autophagy activation via upregulation of autophagy-related gene 7 (*ATG7*) expression, whereas autophagy inhibition enhanced apatinib-induced apoptosis in human GC. miRNA sequencing (miRNA-seq) and circRNA sequencing (circRNA-seq) of the GC tumor xenografts from the control and apatinib groups were performed to further study the mechanism. The downregulated miRNAs of the apatinib group identified by miRNA-seq were further validated by quantitative reverse-transcriptase PCR (qRT-PCR). Our results demonstrated that dysregulated *miR-3657* might contribute to apatinib-induced *ATG7* upregulation. With circRNA-seq and bioinformatics analyses, we demonstrated that *circRACGAP1* might act as an endogenous sponge for *miR-3657* to inhibit its activity. Moreover, under apatinib treatment, *circRACGAP1* was upregulated and triggered autophagy via decreasing *miR-3657* and increasing *ATG7* levels in GC cells and xenografts. Furthermore, silencing of *circRACGAP1* inhibited autophagy and promoted apatinib-induced apoptosis in vitro and in vivo. These findings provided the first evidence that the *circRACGAP1*-*miR-3657-ATG7* axis mediates a regulatory pathway critical for the regulation of autophagy and apatinib sensitivity in GC. In addition, the correlation analysis among the expression of *circRACGAP1*, *miR-3657*, and *ATG7* in GC patients verified the in vitro and in vivo results. Thus, specific blockage of *circRACGAP1* could be a potential therapeutic target for autophagy inhibition in the context of apatinib use in GC.

## Methods

### Cell lines and culture

The human GC cell lines BGC-823 and HGC-27 were purchased from the Shanghai Institute of Biochemistry and Cell Biology, Chinese Academy of Sciences (Shanghai, China). Cells were cultured in RPMI 1640 medium (Gibco Life Technologies, Grand Island, NY, USA) supplemented with 10% fetal bovine serum, 100 U/ml penicillin, and 100 μg/ml streptomycin. The cells were incubated in a humidified atmosphere under 5% CO_2_ at 37 °C.

### Drug preparations and reagents

Apatinib (Selleck Chemicals, Houston, TX, USA) was dissolved in 100% dimethyl sulfoxide (DMSO; Sigma-Aldrich, St Louis, MO, USA) and then diluted with culture medium to the desired concentrations. DMSO added in the treatment group was equal to that in the control group with a final DMSO concentration <0.2% (v/v). Chloroquine were purchased from Sigma-Aldrich (St Louis, MO, USA).

### Plasmids and transfections

The siRNAs specific for ATG7 and *circRACGAP1*, *miR-3567* mimics, and *miR-3567* inhibitors were synthesized by RiboBio (Guangzhou, China). The mRFP-GFP-LC3 plasmid was used to monitor autophagy flux as previously reported^31^. ATG7 plasmid and pcDNA3.1 plasmid were purchased from HanBio (Shanghai, China). Transfections were performed using Lipofectamine 3000 (Invitrogen, Carlsbad, CA, USA) or DharmaFECT 4 (Thermo Scientific, Lafayette, CO, USA), according to the manufacturer’s protocol.

### Clonogenic assay

BGC-823 cells or HGC-27 cells were seeded in 6-well plates (300 cells per well) and incubated overnight. Then, the cells were treated with apatinib at indicated concentrations for 24 h and further cultured in no-drug medium for 2 weeks. For colony scoring, the cells were stained with crystal violet (Beyotime Biotechnology, Nantong, China).

### Cytotoxicity assay and apoptosis assay

The cells were seeded at 5000 cells per well in 96-well plates and incubated overnight. After a particular treatment, the cell viability was determined using Cell Counting Kit-8 (Dojindo, Japan), according to the manufacturer’s instructions. The cell survival rates are expressed as the means ± SD from three independent experiments.

Apoptosis was examined by flow cytometric analysis. The cells were treated with certain concentrations of apatinib for the indicated durations. Both floating and adherent cells were collected, stained with Annexin V–fluorescein isothiocyanate (FITC), and propidium iodide (Dojindo, Kumamoto, Japan), and further analyzed with a flow cytometer (FACScan, BD Biosciences, San Jose, CA, USA) equipped with Cell Quest software (BD Biosciences). Apoptosis was also determined using the TUNEL (Terminal deoxynucleotidyl transferase dUTP nick end labeling) apoptotic cell detection kit (Roche, Basel, Switzerland), according to the manufacturer’s instructions. Apoptosis was expressed as the mean ± SD from three independent experiments.

### Xenografts in mice

Female nude mice (6 weeks old) were purchased from Nanjing Biomedical Research Institute of Nanjing University (Nanjing, China) and maintained under specific pathogen-free conditions. The tumor xenograft models were conducted in nude mice bearing BGC-823 cells (model 1) or BGC-823 cells stably transfected with *circRACGAP1* short hairpin RNA (shRNA) or control shRNA lentivirus (ViGene Biosciences, Rockville, MD, USA) (model 2). In total, 4 × 10^6^ BGC-823 cells were subcutaneously injected into the right axilla of nude mice. When palpable tumors formed, the mice were separately randomized into each group. Then the mice were orally administered control vehicle or 50 mg/kg apatinib daily. Tumor volume was monitored every other day (volume = width^2^ × length × 1/2) for the duration of the experiment. Tumors were collected and weighed at the end of the experiment, and photos were taken at the same time. Six tumor tissues (C_1, C_2, and C_3 from the control group; A_1, A_2, and A_3 from the apatinib group) from model 1 were sent for miRNA-seq and circRNA-seq. Student’s *t*-test was performed to assess the differences between the two groups. All experiments were carried out according to the National Institutes of Health Guidelines for the Care and Use of Laboratory Animals (NIH Publication Number 80–23) revised in 1996.

### Quantitative reverse-transcriptase PCR

Total RNA was extracted from cell cultures by using the Trizol reagent (Gibco Life Technologies, Grand Island, NY, USA), according to the manufacturer’s instructions. The cDNA was amplified with the primers listed in Supplementary Table S1. For miRNA quantification, Bulge-loopTM miRNA qRT-PCR Primer Sets (one RT primer and a pair of qPCR primers for each set) specific for *miR-3657* and *U6* were designed by RiboBio. Divergent primes were used for circRNAs to detect backsplice junctions and convergent primers were used for linear mRNAs. The qRT-PCR analysis was performed using AceQ qPCR SYBR Green Master Mix or miRNA Universal SYBR qPCR Master Mix (Vazyme Biotech Co., Piscataway, NJ, USA) in an ABI Prism 7900 Sequence detection system (Applied Biosystems, Canada). The following thermal cycling conditions were used: 10 min at 95 °C, followed by 40 cycles of denaturation at 95 °C for 15 s and 60 °C for 1 min. Before calculation using the ^ΔΔ^Ct method, the levels of glyceraldehyde 3-phosphate dehydrogenase (GAPDH) and 18S RNA were used to normalize the relative expression levels of mRNA and circRNA, and the levels of small nuclear U6 were used to normalize the miRNA expression levels. Student’s *t*-test was performed to assess the differences between different groups.

### Western blotting, immunohistochemical staining, and antibodies

Western blotting and immunohistochemical staining were performed as previously described^32^. The antibodies used were as follows: monoclonal anti-GAPDH (loading control) (1:1000, Beyotime, Haimen, Jiangsu, China) and monoclonal anti-LC3B, SQSTM1, ATG7, and VEGFR-2 (1: 1000, Cell Signaling Technology, Danvers, MA, USA).

### Confocal microscopy

Cells transfected with mRFP-GFP-LC3 plasmid were seeded in 35 mm glass-bottom dishes and cultured overnight, followed by treatment with apatinib or control vehicle. After the indicated times, the cells were washed with phosphate-buffered saline (PBS) and fixed with 4% paraformaldehyde for 10 min at room temperature. Then, the cells were washed with PBS and left in the dark before fluorescence microscopy analysis. Confocal images of cells were sequentially acquired with Zeiss AIM software on a Zeiss LSM 700 confocal microscope system (Carl Zeiss Jena, Oberkochen, Germany). The autophagosomes are shown as yellow dots (RFP^+^GFP^+^), whereas the autolysosomes are shown as red dots (RFP^+^GFP^−^).

### RNA fluorescence in situ hybridization

Cy3-labeled specific probes to *circRACGAP1* were synthesized by RiboBio. FITC-labeled specific probes to *miR-3657* were synthesized by Sangon Biotech (Shanghai, China). The signals of the probes were detected by the FISH Kit (RiboBio), according to the manufacturer’s instructions.

### Transmission electron microscopy

For the electron microscopy observation, the tumor xenograft tissues or tumor cells were fixed in 2.5% glutaraldehyde immediately after removal. Samples were fixed using 1% osmium tetroxide, followed by dehydration with an increasing concentration gradient of ethanol and propylene oxide. Samples were then embedded in TAAB Epon (Marivac Canada, Inc., St Laurent, Canada), cut into 50 nm sections, and stained with 3% uranyl acetate and lead citrate. Images were acquired using a JEM-1010 electron microscope (JEOL, Tokyo, Japan).

### miRNA-seq and circRNA-seq

Following RNA isolation, the RNA was quantified (Qubit^®^ RNA Assay Kit in Qubit^®^ 3.0 Fluorometer; Life Technologies, CA, USA) and the quality was assessed (RNA Nano 6000 Assay Kit of the BioAnalyzer 2100 system; Agilent Technologies, CA, USA). Next, 3 μg total RNA (miRNA-seq) and 1 μg qualified RNA (circRNA-seq) per sample were used as input material for each library preparation. The library preparations were sequenced on an Illumina Hiseq 2500 platform with a 50 bp single-end module (miRNA-seq) and an Illumina Hiseq X Ten platform with a 150 bp paired-end module (circRNA-seq). Then, the raw reads were filtered for subsequent analysis. CircRNA prediction was performed with circRNA Finder^33^ (https://github.com/bioxfu/circRNAFinder). The miRNA target genes and the circRNA–miRNA interactions were predicted using TargetScan^34^ (http://www.targetscan.org/) and miRanda^35^ (http://www.microrna.org/), and the intersection or union of target genes were treated as the final target genes.

### Sanger sequencing

Sanger sequencing was applied to determine the full length of the amplification products. The divergent primers (Sangon Biotech, Shanghai, China) were designed to confirm the backsplice junction of *circRACGAP1*. The distinct product of the expected size was amplified by outward-facing primers of *circRACGAP1* and was confirmed by Sanger sequencing (Tsingke, Nanjing, China).

### RNA immunoprecipitation

The MagnaRIP RNA-Binding Protein Immunoprecipitation Kit (Millipore, MA, USA) was used for RNA immunoprecipitation (RIP) assay, according to the manufacturer’s instructions. The cell lysates were incubated with beads coated with 5 μg antibody against Argonaute-2 (AGO2) (Abcam, MA, USA) or control rabbit IgG with rotation at 4 °C overnight. Then, total RNA was retrieved and the expression of circRNAs and miRNAs was detected by qRT-PCR analysis.

### Dual-luciferase reporter assay

The luciferase reporter plasmids encoding wild-type genes (*ATG7* 3′UTR-Wt, *circRACGAP1* Wt) and mutated ones (*ATG7* 3′UTR-Mut, *circRACGAP1* Mut) were produced by Geneseed Biotech (Guangzhou, China). The binding sites of *ATG7* and *circRACGAP1* were inserted into the Xhol and NotI sites of the psiCHECK2.0 plasmid (Promega, Madison, WI, USA) in the dual-luciferase reporter assay. First, cells were plated on 24-well plates. Then, 500 ng luciferase reporter plasmid, 50 nM *miR-3657* mimics and negative control were transfected into cells by applying Lipofectamine 3000. Cells were collected and analyzed following the manufacturer’s instructions by using the dual-luciferase assay (Promega, Madison, WI, USA) after 48 h of transfection. All experiments were repeated three times independently.

### Tissue collection

We obtained ten GC tissues from patients who were diagnosed with GC based on histopathological evaluation and underwent surgery at the First Affiliated Hospital of Nanjing Medical University. The study was approved by the Ethics Committee on Human Research of the First Affiliated Hospital of Nanjing Medical University and written informed consent was obtained from all patients.

### Statistical analysis

Data are expressed as the means ± SD. The statistical significance of the differences between the cell lines was analyzed by the parametric unpaired Student’s *t*-test (two-sided). The correlation of the expressions of *circRACGAP1*, *miR-3657*, and *ATG7* were established by Pearson’s correlation analysis. Differences were considered statistically significant when *P* < 0.05.

## Results

### Apatinib inhibits the growth and promotes the apoptosis of GC cells in vitro and in vivo

**Fig. 1:**
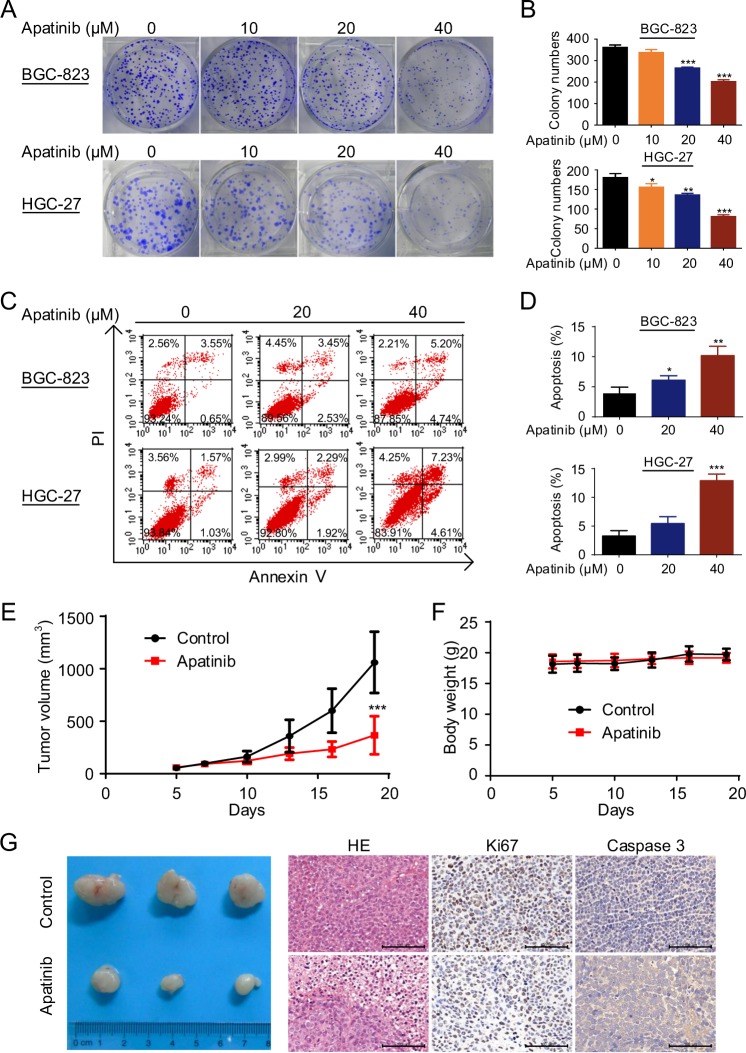
Apatinib inhibits the growth and promotes the apoptosis of GC cells in vitro and in vivo. **a**, **b** Colony-formation assay of BGC-823 cells and HGC-27 cells after apatinib treatment for 24 h. Quantification of numbers of colonies in BGC-823 cells and HGC-27 cells; each colony containing >50 cells was counted. **c**, **d** BGC-823 cells and HGC-27 cells were incubated with apatinib at various concentrations for 48 h. Then, apoptosis induced by apatinib was detected by Annexin V–FITC/PI staining and analyzed using flow cytometry. **e**–**g** Effects of apatinib on the growth of GC in vivo. Statistical analyses of tumor volumes (**e**) and body weights (**f**) in the different groups. Photograph of tumors, hematoxylin–eosin (HE) staining and immunohistochemical staining of tumor tissues (**g**) in mice treated with control vehicle or apatinib (*n* = 6 per subgroup). Bar scale, 100 μm. *P < 0.05; **P < 0.01; ***P < 0.001.

Colony-formation assays of BGC-823 cells and HGC-27 cells were conducted after 24 h of apatinib treatment (Fig. 1a, b). The results demonstrated that apatinib inhibited the proliferation of the two cell lines in a concentration-dependent manner. Flow cytometry assays confirmed the apoptosis induced by apatinib in BGC-823 cells and HGC-27 cells (Fig. 1c, d).

To evaluate the effectiveness of apatinib in vivo, we established tumor xenograft models in nude mice using BGC-823 cells. The mice were orally treated with control vehicle or 50 mg/kg apatinib daily for 14 days. The results showed that apatinib reduced the tumor volumes in the GC xenograft model (Fig. 1e–g). More necrosis was found in tumor sections of apatinib-treated mice compared with those of vehicle-treated mice by histological examination. In addition, immunohistochemical staining showed increased caspase 3 expression together with decreased Ki67 expression in the tumor xenograft tissues of the apatinib group compared with the expression in the tissues of the control vehicle group (Fig. 1g). Collectively, these results indicated that apatinib inhibits the growth and promotes the apoptosis of GC cells in vitro and in vivo.

### Apatinib promotes GC autophagy activation in vitro and in vivo

**Fig. 2:**
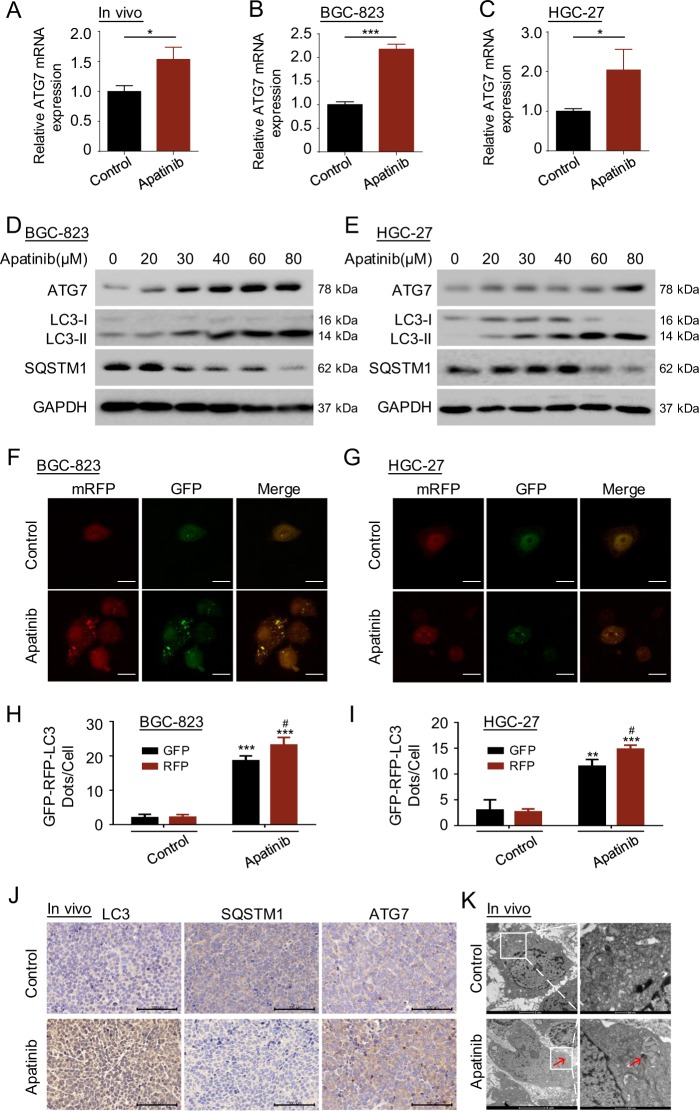
Apatinib promotes GC autophagy activation in vitro and in vivo. **a**–**c** qRT-PCR was performed in vivo (**a**) and in BGC-823 cells (**b**) and HGC-27 cells (**c**) cultured with apatinib. *P < 0.05; ***P < 0.001. **d**, **e** Western blotting was performed on autophagy proteins expressed in BGC-823 cells (**d**) and HGC-27 cells (**e**) co-cultured with apatinib at various concentrations for 48 h. **f**, **g** BGC-823 cells (**f**) and HGC-27 cells (**g**) were transfected with mRFP-GFP-LC3 plasmid, followed by incubation with apatinib. Confocal microscopy analysis is shown. Bar scale, 10 μm. **h**, **i** The numbers of GFP+ or RFP+ dots per cell are presented as the means ± SD of three independent experiments. **P < 0.01; ***P < 0.001 compared with the corresponding control group. ^#^*P* < 0.05 compared with the GFP group treated with apatinib. **j** Immunohistochemical staining of LC3-II, SQSTM1, and ATG7 in the tumor xenograft tissues from the apatinib and control groups. Bar scale, 100 μm. **k** Autophagosomes were observed by TEM in tumor xenografts from the apatinib group. Bar scale, 500 nm (right, enlarged).

The mechanisms by which apatinib promotes GC cell apoptosis have been reported in some studies^11,36,37^. However, the relationship between autophagy and apatinib and the mechanisms are not clear. To examine the potential relationship between autophagy and apatinib, we detected the expression of autophagy genes in vitro and in vivo. First, qRT-PCR analysis of the *ATG* genes were conducted in BGC-823 cells after apatinib treatment (Supplementary Fig. S1B). Apatinib increased the expression of multiple autophagy genes, with the change in the expression of the *ATG7* gene being the most significant. Next, we confirmed that apatinib affected the *ATG7* mRNA levels in the tumor tissues of BGC-823 cell-mediated xenografts (Fig. 2a) and in GC cell lines (Fig. 2b, c). These data suggested that apatinib might drive autophagy activation in GC. To examine this possibility, we performed autophagy functional assays in GC cells co-cultured with apatinib. Apatinib increased ATG7 protein expression and the LC3-II protein expression, as well as decreased sequestosome 1 (SQSTM1) expression in BGC-823 cells and HGC-27 cells (Fig. 2d, e and Supplementary Fig. S1C) in a concentration-dependent manner. Moreover, apatinib-induced autophagic flux in BGC-823 cells and HGC-27 cells (Fig. 2f–i) transiently transfected with mRFP-GFP-LC3 plasmid. In addition, in the BGC-823 cell-mediated xenograft model, immunohistochemical staining showed increased LC3-II and ATG7 expression together with decreased SQSTM1 expression in the tumor tissues of the apatinib group compared with the expression in the tumor tissues of the control vehicle group (Fig. 2j). Transmission electron microscopy (TEM) assay showed the formation of autophagic vesicles in the tumor xenograft tissues from the apatinib group (Fig. 2k). Thus, our data indicated that apatinib promotes GC autophagy activation in vitro and in vivo.

### Silencing of ATG7 promotes apatinib-induced apoptosis in GC cells

**Fig. 3:**
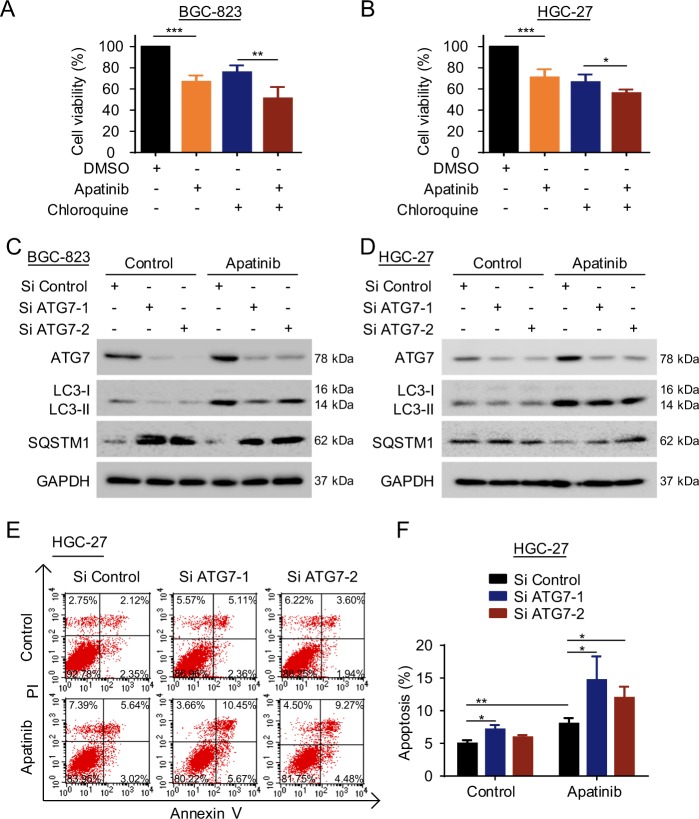
Silencing of ATG7 promotes apatinib-induced apoptosis in GC cells. **a**, **b** CCK8 assays were conducted in BGC-823 cells (**a**) and HGC-27 cells (**b**) co-cultured with apatinib (40 µM apatinib in BGC-823 cells; 10 µM apatinib in HGC-27 cells) and 20 µM chloroquine for 24 h. **c**, **d** The expression of autophagy proteins was detected by western blotting in BGC-823 cells (**c**) and HGC-27 cells (**d**) transfected with ATG7 siRNA and incubated with 40 µM apatinib for 48 h. **e**, **f** Apoptosis was detected by flow cytometry in HGC-27 cells. The cells were transfected with ATG7 siRNA and subsequently exposed to 40 µM apatinib for 30 h. *P < 0.05; **P < 0.01; ***P < 0.001.

We next examined the potential role of the autophagy pathway in apatinib sensitivity in GC cells. An autophagy lysosomal inhibitor, chloroquine, was used with or without apatinib to treat HGC-27 cells and BGC-823 cells. Combination of chloroquine and apatinib significantly inhibited cancer cells proliferation (Supplementary Fig. S2A and Fig. 3a, b). Moreover, the cell proliferation inhibition induced by apatinib was promoted by silencing of ATG7 in BGC-823 cells (Supplementary Fig. S2B). Knockdown of ATG7 decreased the LC3-II expression, increased SQSTM1 expression (Fig. 3c, d and Supplementary Fig. S2C, D), and promoted the BGC-823 cell and HGC-27 cell apoptosis induced by apatinib (Supplementary Fig. S2E and Fig. 3e, f). Collectively, the data strongly suggested that apatinib activates the autophagy pathway, whereas downregulation of the expression of the ATG7 promotes apatinib sensitivity in GC cells.

### Apatinib activates ATG7 via downregulation of miR-3657

**Fig. 4:**
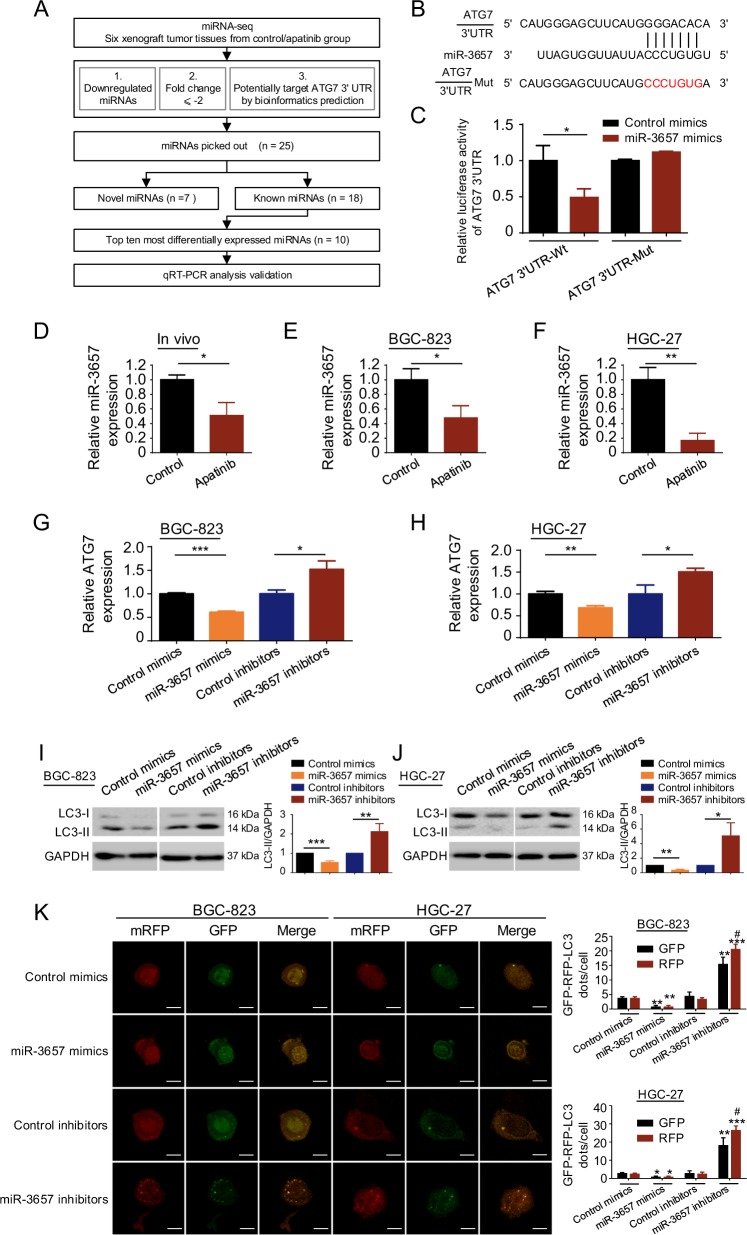
Apatinib activates ATG7 via downregulation of *miR-3657*. **a** The flowchart shows the inclusion criteria for the downregulated miRNAs potentially targeting *ATG7* from the miRNA-seq analysis. **b** The predicted binding sequences for *miR-3657* within the human *ATG7* 3′-UTR by TargetScan. The mutation sequences of *ATG7* Mut are shown in red. **c** Luciferase activity was measured in BGC-823 cells transfected with *miR-3657* mimics or control miRNA and *ATG7* 3′UTR-Wt or *ATG7* 3′UTR-Mut. Luciferase activity was normalized to that in cells transfected with control miRNA. **d**–**f** qRT-PCR was performed in vivo (**d**) and in BGC-823 cells (**e**) and HGC-27 cells (**f**) incubated with apatinib, to detect the expression of *miR-3657*. **g**, **h** qRT-PCR was performed in BGC-823 cells (**g**) and HGC-27 cells (**h**), to detect the expression of *ATG7* after transfection with *miR-3657* mimics or inhibitors. **i**, **j** The protein level of LC3-II was detected by western blotting in BGC-823 cells (**i**) and HGC-27 cells (**j**) transfected with *miR-3657* mimics or inhibitors. The LC3-II protein level was quantified by densitometry and normalized to GAPDH, to reflect the LC3-II/GAPDH expression ratio. *P < 0.05; **P < 0.01. **k** BGC-823 cells and HGC-27 cells were transfected with mRFP-GFP-LC3 plasmid and *miR-3657* mimics or inhibitors. Confocal microscopy analysis is shown. Bar scale, 10 μm. The numbers of GFP+ or RFP+ dots per cell are presented as the means ± SD of three independent experiments. *P < 0.05; **P < 0.01; ***P < 0.001 compared with the corresponding control group. ^#^*P* < 0.05 compared with the GFP group transfected with *miR-3657* inhibitors.

miRNAs often play significant gene-regulatory roles by pairing with target mRNAs and repressing their expression^38^. We hypothesized that dysregulated miRNAs might contribute to the increase in ATG7 expression. To test this hypothesis, miRNA-seq was performed in six tumor xenograft tissues from the BGC-823 cell-mediated xenograft model in Fig. 1g. Figure 4a shows the criteria we used to pick out the top ten most differentially downregulated miRNAs that might target *ATG7*. A qRT-PCR assay was conducted to confirm the expression of the ten downregulated miRNAs and *miR-3657* was the most differentially expressed (Supplementary Fig. S3A). Bioinformatics analyses with TargetScan (Fig. 4b) and miRanda (Supplementary Fig. S3B) revealed that *miR-3657* most likely targeted the 3′-untranslated region (3′-UTR) regions of the *ATG7* gene. The intersection of sequences obtained by the two miRNA target prediction softwares was proposed to be a potential binding site. Then, wild-type and mutant *ATG7* reporter plasmids targeting the identified sequence were designed (Fig. 4b). Luciferase reporter assays demonstrated that *miR-3657* suppressed luciferase activity in BGC-823 cells transfected with wild-type *ATG7* reporter plasmid but not in those transfected with the mutant one (Fig. 4c). *MiR-3657* expression was decreased in apatinib-treated GC xenograft tissues and GC cells compared with that in the control conditions (Fig. 4d–f). In addition, qRT-PCR showed that *miR-3657* mimics decreased the mRNA levels of *ATG7* and *ATG7* mRNA expression was rescued by *miR-3657* inhibitors in BGC-823 cells and HGC-27 cells (Fig. 4g, h). Also, western blotting confirmed the protein levels of LC3-II regulated by *miR-3657* (Fig. 4i, j). Confocal microscopy analysis demonstrated that *miR-3657* inhibitors induced the autophagic flux, whereas *miR-3657* mimics decreased the number of autophagosomes in BGC-823 cells and HGC-27 cells transiently transfected with mRFP-GFP-LC3 plasmid (Fig. 4k). Thus, these data indicated that apatinib increases ATG7 expression via a selective loss of *miR-3657*.

### CircRNA-seq of the tumor xenograft tissues from the apatinib group and control group

**Fig. 5:**
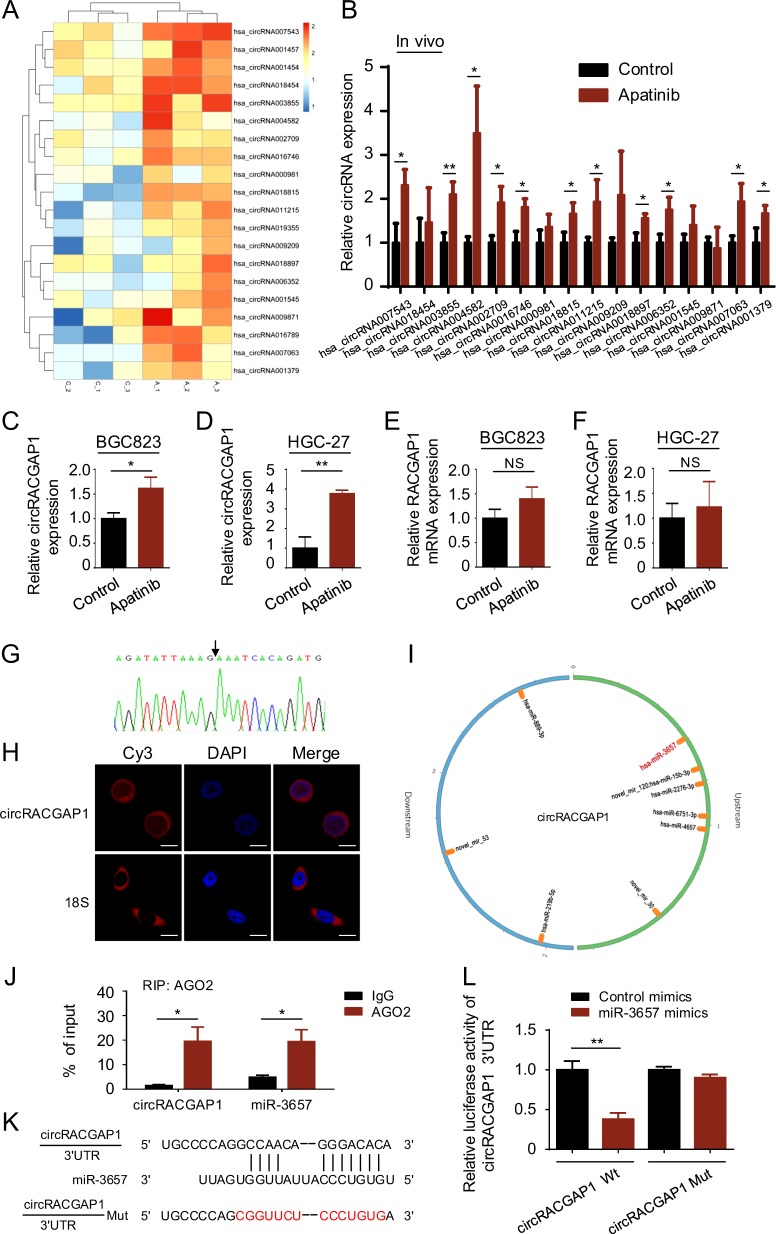
*CircRACGAP1* acts as an endogenous sponge for *miR-3657*. **a** RNA-seq analysis of circRNAs was performed in three paired tumor xenografts from the control vehicle group and apatinib group. **b** qRT-PCR was performed in vivo to confirm the circRNA-seq results. **c**–**f** qRT-PCR was used in BGC-823 cells and HGC-27 cells cultured with apatinib to detect the expression of *circRACGAP1* (**c**, **d**) and *RACGAP1* (**e**, **f**). NS: not significant. **g** *CircRACGAP1* was validated by Sanger sequencing. **h** RNA FISH was performed to detect the localization of *circRACGAP1* in BGC-823 cells. 18S rRNA was used as a positive control for the cytoplasmic fractions. Bar scale, 10 μm. **i** A schematic model showing the putative binding sites of miRNAs (fold change ≥2 or ≤ −2) and *circRACGAP1* using miRanda. **j** RNA immunoprecipitation (RIP) for AGO2 in BGC-823 cells was performed. *CircRACGAP1* and *miR-3657* expression levels were detected by qRT-PCR. **k** The predicted binding sequences for *miR-3657* within the human *circRACGAP1* 3′UTR by TargetScan. The mutation sequences of *circRACGAP1* Mut are shown in red. **l** Luciferase activity was measured in BGC-823 cells transfected with *miR-3657* mimics or control miRNA, and *circRACGAP1* Wt or *circRACGAP1* Mut. The luciferase activity was normalized to that in cells transfected with control miRNA. *P < 0.05; **P < 0.01.

We next investigated the mechanisms by which apatinib upregulates *ATG7* and downregulates *miR-3657*. CircRNAs have been reported to act as competing endogenous RNA (ceRNA) sponges, interacting with miRNAs and influencing their activity^30^. Thus, we proposed the following hypothesis: certain circRNAs might function as a *miR-3657* sponge and further regulate *ATG7* expression. Hence, we performed circRNA-seq analysis to identify differentially expressed circRNAs between the control group and apatinib group in the BGC-823 cell-mediated xenograft model. In all samples, 19,904 distinct circRNA candidates were found, of which 3798 circRNAs have been found in circBase (Supplementary Fig. S4A). The Venn diagrams illustrate the relationships among the six sequencing samples of the circRNAs (Supplementary Fig. S4B, C). Considering the circRNA characteristics and ease of validation in future studies, circRNAs that met the following criteria were included in this study: exonic circRNAs expressed in every sequencing sample with a length <1000 nucleotides^23^. The differentially expressed circRNAs are shown in the heat map (Fig. 5a).

Then we confirmed the circRNA-seq results of these circRNAs from Fig. 5a in the tumor xenograft tissues by qRT-PCR analysis (Fig. 5b). This analysis confirmed one of the most differentially expressed circRNAs (*hsa_circRNA004582*, chr12: 49991998–49992655). The corresponding parent gene of *hsa_circRNA004582* is *RACGAP1* (*Rac GTPase activating protein 1*). Thus, we termed this circRNA “*circRACGAP1*”. A qRT-PCR assay demonstrated that *circRACGAP1* mRNA expression was upregulated in apatinib-treated GC cells (Fig. 5c, d). In addition, we examined the mRNA level of *RACGAP1* in GC cells treated with apatinib. The results showed no difference in the mRNA expression of *RACGAP1* between control and apatinib-treated GC cells (Fig. 5e, f). Therefore, the results excluded the hypothesis that *miR-3657* might bind the corresponding parent gene of *circRACGAP1*.

Figure 5g shows the circular junction of *circRACGAP1* confirmed by Sanger sequencing. RT-PCR with divergent primers detected *circRACGAP1* in cDNA but not in genomic DNA (Supplementary Fig. S4D). The fluorescence in situ hybridization (FISH) indicated that *circRACGAP1* mainly resided in the cytoplasm in BGC-823 cells (Fig. 5h). Taken together, these results confirmed the most differentially expressed circRNA-*circRACGAP1* identified by circRNA-seq.

### CircRACGAP1 acts as an endogenous sponge for miR-3657

Next, we constructed a circRNA–miRNA network (Supplementary Fig. S5) to visualize their interactions based on our circRNA-seq data and miRNA-seq data using bioinformatics analyses. A schematic model shows the possible binding sites of *circRACGAP1* on miRNAs (fold change ≥2 or ≤−2) from the miRNA-seq analysis by miRanda, and the possible binding site of *circRACGAP1* on *miR-3657* is shown in red (Fig. 5i). To examine whether *miR-3657* could bind to *circRACGAP1*, RIP for AGO2 in BGC-823 cells was performed, indicating that *circRACGAP1* and *miR-3657* were substantially accumulated in the AGO2 pellet (Fig. 5j). Bioinformatics analyses with bioinformatics tools TargetScan (Fig. 5k) and miRanda (Supplementary Fig. S4E) predicted that *miR-3657* might target the 3′-UTR of *circRACGAP1*. Furthermore, luciferase reporter assays demonstrated that *miR-3657* suppressed the luciferase activity in BGC-823 cells transfected with wild-type *circRACGAP1* reporter plasmid but not in those transfected with the mutant one (Fig. 5l). The immunofluorescence experiment confirmed the co-localization of *circRACGAP1* and *miR-3657* (Supplementary Fig. S4F). Thus, these results indicated that *miR-3657* binds *circRACGAP1*.

### CircRACGAP1 modulates apatinib-induced autophagy via targeting miR-3657 and ATG7

**Fig. 6:**
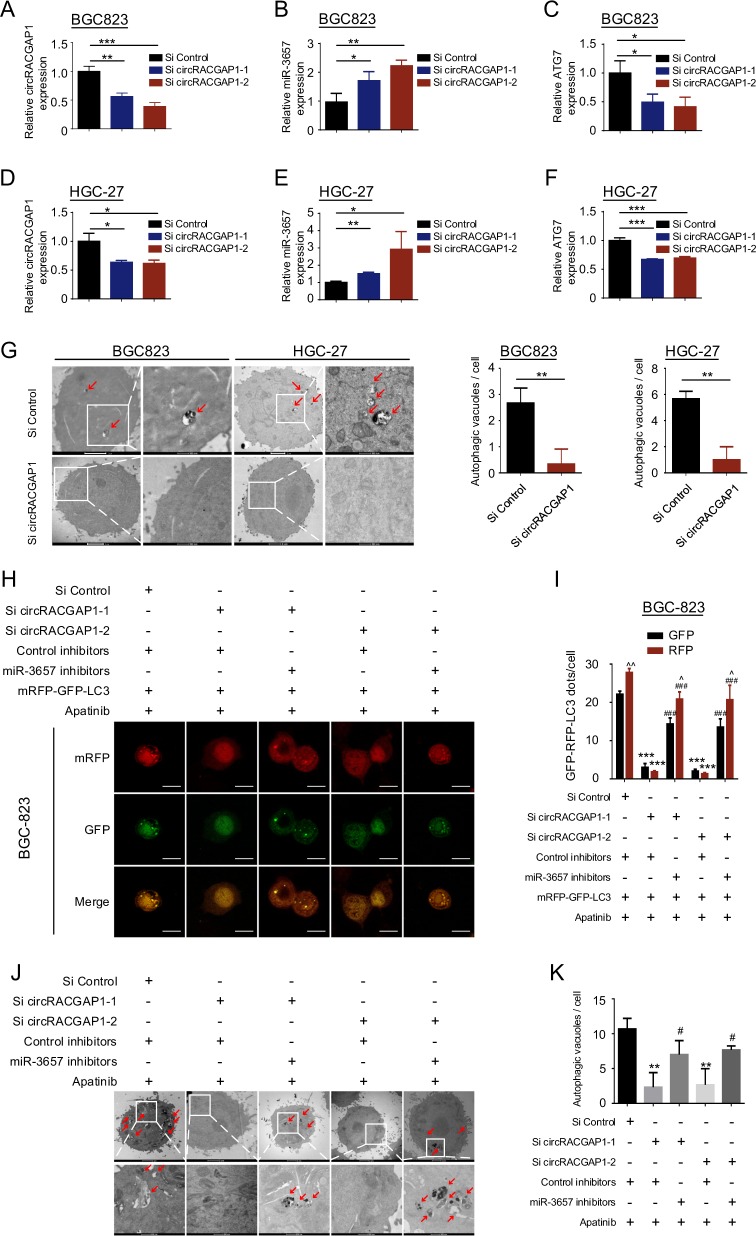
*CircRACGAP1* modulates apatinib-induced autophagy via targeting *miR-3657* and *ATG7*. **a**–**f** qRT-PCR was performed in BGC-823 cells (**a**–**c**) and HGC-27 cells (**d**–**f**) to detect the expression of *circRACGAP1*, *miR-3657*, and *ATG7* after transfection with *circRACGAP1* siRNA. **g** Autophagosomes were observed by TEM in BGC-823 and HGC-27 cells transfected with si *circRACGAP1*-1 and control siRNA. Bar scale, 500 nm (enlarged). *P < 0.05; **P < 0.01; ***P < 0.001. **h**, **i** BGC-823 cells were cotransfected with *circRACGAP1* siRNA or control siRNA, *miR-3657* inhibitors or control inhibitors, and mRFP-GFP-LC3 plasmid. After 24 h, the cells were incubated with 40 µM apatinib for 24 h and analyzed by confocal microscopy. Bar scale, 10 μm. The numbers of GFP+ or RFP+ dots per cell are presented as the means ± SD of three independent experiments. ***P < 0.001 compared with the group transfected with si control. ###*P* < 0.001 compared with the group cotransfected with si *circRACGAP1*-1 or si *circRACGAP1*-2 and control inhibitors. ^*P* < 0.05; ^^*P* < 0.01 compared with the corresponding GFP group. **j**, **k** Autophagic vacuoles were observed by TEM in BGC-823 cells cotransfected with *circRACGAP1* siRNA or control siRNA, and *miR-3657* inhibitors or control inhibitors, and followed by incubation with 40 µM apatinib for 24 h. **P < 0.01 compared with the group transfected with si control. #*P* < 0.05 compared with the group cotransfected with si *circRACGAP1*-1 or si *circRACGAP1*-2 and control inhibitors.

With miRNA-seq and circRNA-seq, we found that *circRACGAP1* might function as an endogenous sponge for *miR-3657* and *ATG7*. A qRT-PCR assay demonstrated that silencing of *circRACGAP1* increased *miR-3657* expression and decreased *ATG7* mRNA levels in BGC-823 cells (Fig. 6a–c) and HGC-27 cells (Fig. 6d–f). Moreover, the TEM assay showed that transfection with *circRACGAP1* siRNA reduced the number of autophagosomes in BGC-823 and HGC-27 cells (Fig. 6g).

Apatinib-induced activation of *circRACGAP1*, which functions as a ceRNA sponge for *miR-3657* and *ATG7*, led us to hypothesize that *circRACGAP1* might regulate autophagy-mediated apatinib resistance. To test this hypothesis, BGC-823 cells and HGC-27 cells were cotransfected with *circRACGAP1* siRNA or control siRNA, *miR-3657* inhibitors or control inhibitors, and mRFP-GFP-LC3 plasmid, and followed by incubation with apatinib. The apatinib-induced autophagosomes and autolysosomes were reduced in *circRACGAP1* knockdown BGC-823 cells and HGC-27 cells (Fig. 6h, i and Supplementary Fig. S6A, B), but rescued by transfection with *miR-3657* inhibitors. In addition, the TEM assay confirmed that transfection with *circRACGAP1* siRNA reduced the number of autophagic vacuoles in apatinib-treated BGC-823 cells, but rescued by transfection with *miR-3657* inhibitors (Fig. 6j, k). Therefore, the data demonstrated that *circRACGAP1* modulates apatinib-induced autophagy via targeting *miR-3657* and *ATG7*.

### Silencing of circRACGAP1 sensitizes GC cells to apatinib in vitro and in vivo via modulating autophagy

**Fig. 7:**
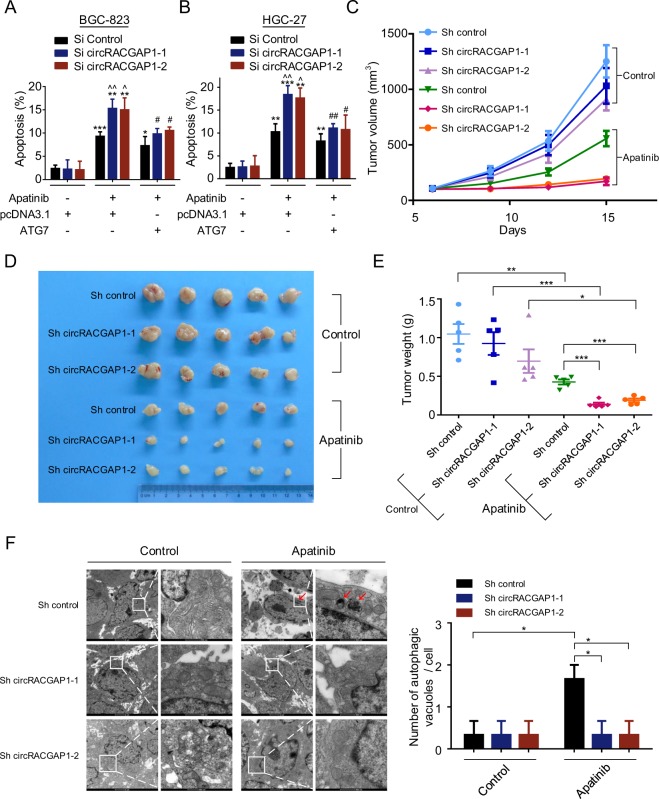
Silencing of *circRACGAP1* sensitizes GC cells to apatinib in vitro and in vivo via modulating autophagy. **a**, **b** Apoptosis was detected in BGC-823 and HGC-27 cells cotransfected with *circRACGAP1* siRNA or control siRNA, and ATG7 plasmid or pcDNA3.1 plasmid by the TUNEL assay. Then, the cells were co-cultured with or without 40 µM apatinib for 30 h. **P < 0.01; ***P < 0.001 compared with the control group without apatinib treatment. ^*P* < 0.05; ^^*P* < 0.01 compared with the apatinib group cotransfected with pcDNA3.1 plasmid and si control. #*P* < 0.05; ##*P* < 0.01 compared with the apatinib group cotransfected with pcDNA3.1 plasmid and si *circRACGAP1*-1 or si *circRACGAP1*-2. **c**–**e** Tumor xenograft model in nude mice bearing BGC-823 cells stably transfected with *circRACGAP1* shRNA lentivirus or control shRNA lentivirus. The mice were randomly divided into control group and apatinib group. Tumor volumes (**c**) and tumor weights (**e**) in the different groups (*n* = 5 per subgroup). Photograph of tumors (**d**) in mice were taken. **f** Autophagosomes were observed by TEM in tumor xenografts. Bar scale, 500 nm (enlarged). *P < 0.05; **P < 0.01; ***P < 0.001.

**Fig. 8:**
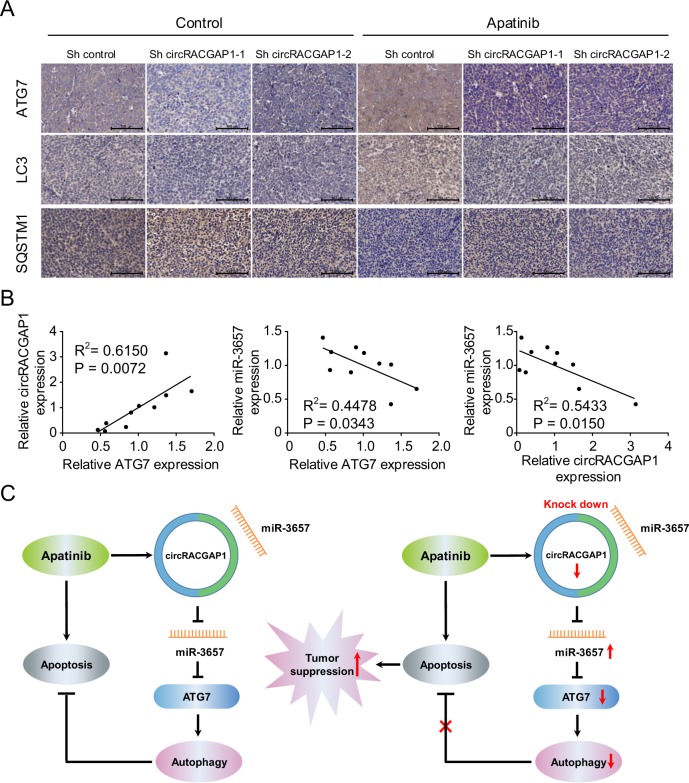
Role of *circRACGAP1* in mediating autophagy and apatinib sensitivity. **a** Immunohistochemical staining of ATG7, LC3-II, and SQSTM1 in the tumor xenograft tissues from the apatinib and control groups. Bar scale, 100 μm. **b** Correlation analysis of relative *ATG7* expression and relative *circRACGAP1* expression (left), relative *ATG7* expression and relative *miR-3657* expression (middle), and relative *circRACGAP1* expression and relative *miR-3657* expression (right) in ten gastric cancer tissues. **c** Schematic diagram of the relationship among apatinib, the *circRACGAP1*-*miR-3657-ATG7* axis, autophagy and apoptosis in GC. Left: apatinib induces autophagy via the *circRACGAP1*-*miR-3657-ATG7* axis. Right: knockdown of *circRACGAP1* promotes the antitumor activity of apatinib via inhibition of autophagy.

Next, we demonstrated that silencing of *circRACGAP1* expression promoted apatinib-induced apoptosis, but rescued by transfection with ATG7 plasmid in GC cells (Fig. 7a, b). In addition, we established a tumor xenograft model in nude mice bearing BGC-823 cells stably transfected with *circRACGAP1* shRNA lentivirus or control shRNA lentivirus. The transfection effect of *circRACGAP1* shRNA lentivirus was confirmed in Supplementary Fig. S7. When palpable tumors formed, the mice were separately randomized into the control group or apatinib group. The results showed that silencing of *circRACGAP1* promoted the antitumor effect of apatinib (Fig. 7c–e). TEM assay demonstrated that silencing of *circRACGAP1* reduced the number of autophagic vacuoles of the xenograft tissues in the apatinib group (Fig. 7f). In addition, immunohistochemical staining of the xenograft tissues showed that transfection with *circRACGAP1* shRNA lentivirus in the apatinib group decreased the expression of ATG7 and LC3-II, and rescued SQSTM1 expression compared with the expression in the tissues of the apatinib group transfected with control shRNA lentivirus (Fig. 8a). Collectively, these results demonstrated that silencing of *circRACGAP1* sensitizes GC cells to apatinib in vitro and in vivo via autophagy inhibition.

Moreover, we examined the *circRACGAP1*, *miR-3657*, and *ATG7* expression in ten GC tissues (Fig. 8b). Positive correlation was observed between *circRACGAP1* and *ATG7* (*P* = 0.0072). Negative correlations were observed between *ATG7* and *miR-3657* (*P* = 0.0343), and between *circRACGAP1* and *miR-3657* in GC tissue (*P* = 0.0150). Thus, the results of *circRACGAP1*-*miR-3657*-*ATG7* axis in GC patients were in accordance with the previous conclusions from GC cell lines and animal experiments.

## Discussion

In this translationally relevant study, we reported for the first time that specific blockage of *circRACGAP1* may be a potential therapeutic strategy to enhance the antitumor effect of apatinib in GC. The results demonstrated that apatinib promotes autophagy activation via upregulation of ATG7 expression and autophagy inhibition promotes apatinib-induced apoptosis in human GC. With miRNA-seq and circRNA-seq analyses, our study provided new insights into the function of *circRACGAP1*. The results showed that *circRACGAP1* acts as an endogenous sponge for *miR-3657* to inhibit its activity and further upregulate the *miR-3657*-targeted gene *ATG7*. Silencing of *circRACGAP1* inhibited apatinib-induced autophagy by downregulation of *ATG7*, which was rescued by *miR-3657*. Moreover, knockdown of *circRACGAP1* sensitizes GC cells to apatinib via autophagy inhibition in vitro and in vivo. These findings provided the first evidence that the *circRACGAP1*-*miR-3657-ATG7* axis mediates a regulatory pathway critical for the regulation of autophagy and apatinib sensitivity in GC (Fig. 8c).

To improve sensitivity to kinase inhibitors, current strategies attempt to target the same kinase in a different way^39^ or target a parallel signaling pathway^40^, whereas the strategy of inhibiting autophagy may represent an entirely independent process of reversing drug resistance^41^. Under most circumstances, autophagy constitutes a stress adaptation that suppresses apoptosis and promotes cell survival^13^. However, autophagy can also kill cells by type II or autophagic cell death, which is a form of nonapoptotic programmed cell death^42^. Interestingly, similar stimuli can induce apoptosis or autophagy and sometimes a mixed phenotype of autophagy and apoptosis. The mechanisms for these different phenotypes are related to the degradation of different pro-apoptotic or anti-apoptotic regulators by autophagy^14,15^. A role of autophagy in protecting cells from undergoing programmed cell death was found in some completed and ongoing phase I/II clinical studies concerning chloroquine and hydroxychloroquine^43^. In addition, inhibition of autophagy by chloroquine or hydroxychloroquine combined with chemotherapy, kinase inhibitors, or radiation therapy has been shown to improve antitumor activity^16–18^. Apatinib was reported to promote apoptosis in osteosarcoma, hepatocellular carcinoma, and intrahepatic cholangiocarcinoma^11,36,37^. However, the relationship between autophagy and apoptosis, and the mechanisms had not yet been reported in GC. Moreover, whether inhibiting autophagy might reverse apatinib resistance in GC and the related mechanisms still remained to be resolved. In this study, we demonstrated that apatinib promoted autophagy activation in GC. Our study is the first to report that autophagy inhibition promoted apatinib-induced apoptosis via downregulation of *ATG7* level in GC. The *ATG7* gene plays a complex role in cancer therapy. Strohecker et al.^44^ reported that *ATG7* initially showed antitumor efficacy at earlier stages of *BRAF*^V600E^-driven lung tumorigenesis via decreasing oxidative stress, whereas *ATG7* promoted tumorigenesis by preserving mitochondrial function at later stages of tumorigenesis. Acute *ATG7* ablation in mice with preexisting non-small cell lung cancer suppressed tumor growth via shutting off the mammalian target of rapamycin and mitogen-activated protein kinase signaling^45^. These studies provided the rationale for targeting *ATG7* in cancer therapy.

Furthermore, we report a novel autophagy regulatory mechanism in mediating tumor cell fate and tumor progression by a circRNA in response to apatinib treatment. CircRNAs are novel biomarkers for human GC, hepatocellular carcinoma development, and oral cancer^46–50^. Moreover, circRNAs participate in autophagy regulatory networks and mediate the transcriptional and posttranscriptional regulation of *ATGs*^28,51^. Huang et al.^52^ reported that *circHIPK2* regulated astrocyte activation via the regulation of autophagy and endoplasmic reticulum stress by targeting *MIR124-2HG* and *SIGMAR1*. Hansen et al.^30^ reported that *ciRS-7* acted as a sponge for *miR-7* and increased the expression levels of *miR-7* target genes. Herein, with miRNA-seq and circRNA-seq technology, we demonstrated that apatinib induces expression of *circRACGAP1*, which interacts with *miR-3657* and as a result, *miR-3657* repression of *ATG7* mRNA is released, eliciting increased autophagy. In addition, silencing of *circRACGAP1* inhibited autophagy and promoted apatinib-induced apoptosis via sponging of *miR-3657* and further decreasing *ATG7* expression in vitro an in vivo. Collectively, the *circRACGAP1*-*miR-3657*-*ATG7* axis mediates a novel regulatory pathway critical for the apatinib sensitivity via regulation of autophagy in GC.

In this study, we proposed that knockdown of *circRACGAP1* may be a potential intervention strategy to reduce the toxicities and promote the antitumor effect of apatinib via autophagy inhibition in GC patients. As circRNAs are stable in blood plasma and exosomes^49,53^, further study of monitoring the changes of plasma circRNAs levels and exosomal circRNAs levels may help distinguish patients with different curative effects. CircRNA biogenesis and expression has been reported to be regulated by different mechanisms: the core spliceosomal machinery, *cis*-elements, and RNA-binding proteins^54–58^. Our results showed that mRNA levels of *RACGAP1* were not significantly different between the control group and apatinib group (Fig. 5e, f). Moreover, we detected the expression of some reported genes involved in circRNA biogenesis. We found that SF3A2 (splicing factor 3A2) was downregulated in apatinib-treated tumor xenograft tissues compared with that without apatinib treatment (Supplementary Fig. S8A), and silencing of SF3A2 increased *circRACGAP1* expression (Supplementary Fig. S8B). In the follow-up study, we will provide more results of how apatinib affect *circRACGAP1* expression and more systematic/in-depth molecular mechanisms.

Our previous study reported that JWA (adenosine diphophate-ribosylation-like factor 6 interacting protein 5, ARL6ip5) reversed cisplatin resistance in human GC^59^, whereas lower expression of JWA sensitized cisplatin-resistant GC cells to lapatinib-induced apoptosis^32^. Collectively, these three studies have led to the proposals for some significant therapy regimens in GC patients: higher expression of JWA may be used as a biomarker for cisplatin treatment, lower expression of JWA may be utilized to identify patients that would benefit from HER2-targeted therapy; in terms of the anti-angiogenesis therapy, silencing of *circRACGAP1* may be a potential intervention strategy to reduce the toxicities and promote the antitumor effect of apatinib. These proposals may help select effective therapy regimens for specific patients and mitigate drug toxicities. Further clinical studies are needed to demonstrate the results for better personalized therapy regimens in GC patients^60^.

In conclusion, we report for the first time that the *circRACGAP1*-*miR-3657-ATG7* axis mediates a regulatory pathway critical for the regulation of autophagy and apatinib sensitivity in GC. Specific blockage of *circRACGAP1* can be utilized as a potential therapeutic target for autophagy inhibition in GC patients undergoing apatinib treatment. Furthermore, we postulate that *circRACGAP1* might contribute to the further development of personalized therapeutics by combining its inhibitors with apatinib against human GC. These findings might provide new opportunities for prospective multi-institutional trials testing clinical applications of *circRACGAP1* as a potential intervention target to promote the antitumor efficacy of apatinib in advanced GC patients.

## Supplementary information

SUPPLEMENTAL MATERIAL

## Data Availability

The datasets used and/or analyzed during the current study are available from the corresponding author on reasonable request.
