# Supplementary material for: Silencing of circRACGAP1 sensitizes gastric cancer cells to apatinib via modulating autophagy by targeting miR-3657 and ATG7
Source: Cell Death Dis. 2020 Mar 5;11(3):169. doi: 10.1038/s41419-020-2352-0 (PMC7058073; doi:10.1038/s41419-020-2352-0)
Supplement: Supplementary file 1 — SUPPLEMENTAL MATERIAL [file 41419_2020_2352_MOESM1_ESM.docx]

**Supplementary Figures**


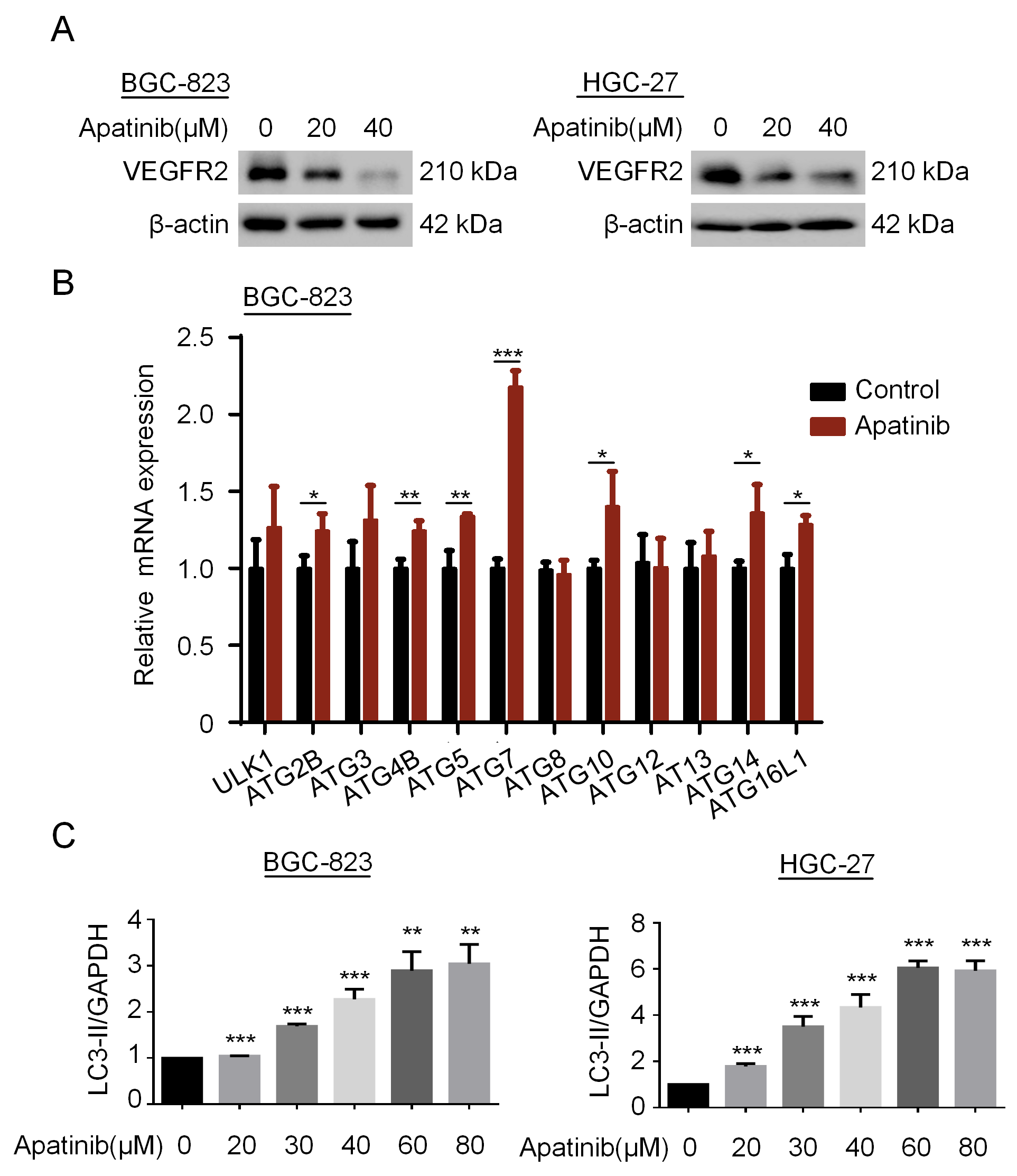


**Figure S1.** Supporting information for Figure 2. Apatinib upregulates the expression of *ATG* genes in BGC-823 cells. (**A**) BGC-823 and HGC-27 cells were treated with 20 µM or 40 µM apatinib for 24 h. VEGFR2 levels were examined by western blot. β-actin as loading control. (**B**) A qRT-PCR assay was performed in BGC-823 cells treated with apatinib to examine *ATG* gene expression. (**C**) The LC3-II proteins levels in Figure 2D and E were quantitated by densitometry and normalized to GAPDH to reflect the LC3-II/GAPDH expression ratio. *, *P* < 0.05; **, *P* < 0.01; ***, *P* < 0.001.


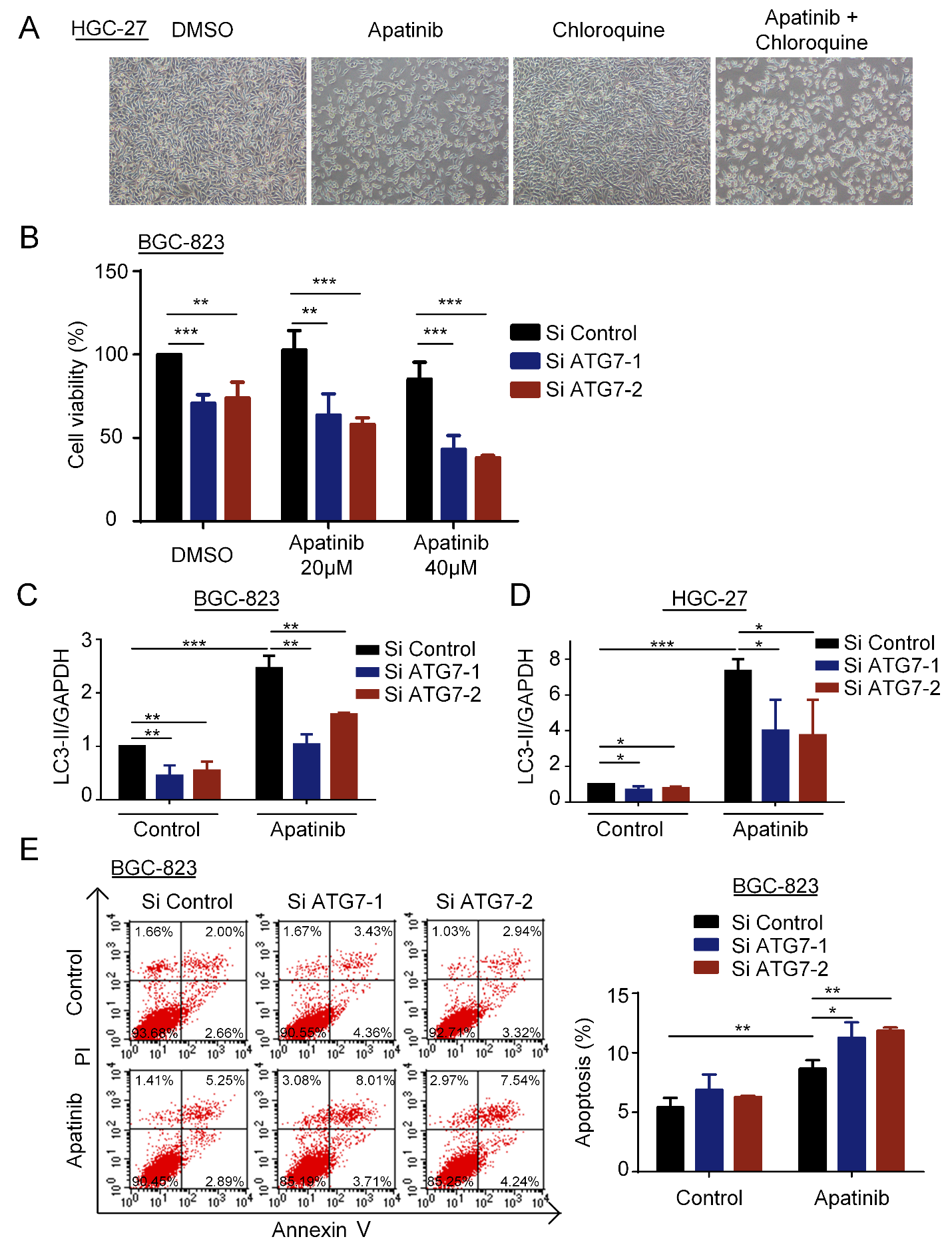


**Figure S2.** Supporting information for Figure 3. Autophagy inhibitor combined with apatinib promotes the inhibition of cell proliferation in GC cells. (**A**) HGC-27 cells were treated with 20 µM chloroquine and 40 µM apatinib. Then, the cells were photographed. (**B**) BGC-823 cells were transfected with ATG7 siRNA, followed by treatment with different concentrations of apatinib for 24 h. Then, CCK8 assay was conducted. (**C and D**) The LC3-II proteins levels in Figure 3 were quantitated by densitometry and normalized to GAPDH to reflect the LC3-II/GAPDH expression ratio. (**E**) Apoptosis was detected by flow cytometry in BGC-823 cells. The cells were transfected with ATG7 siRNA and subsequently exposed to 40 µM apatinib for 30 h. *, *P* < 0.05; **, *P* < 0.01; ***, *P* < 0.001.


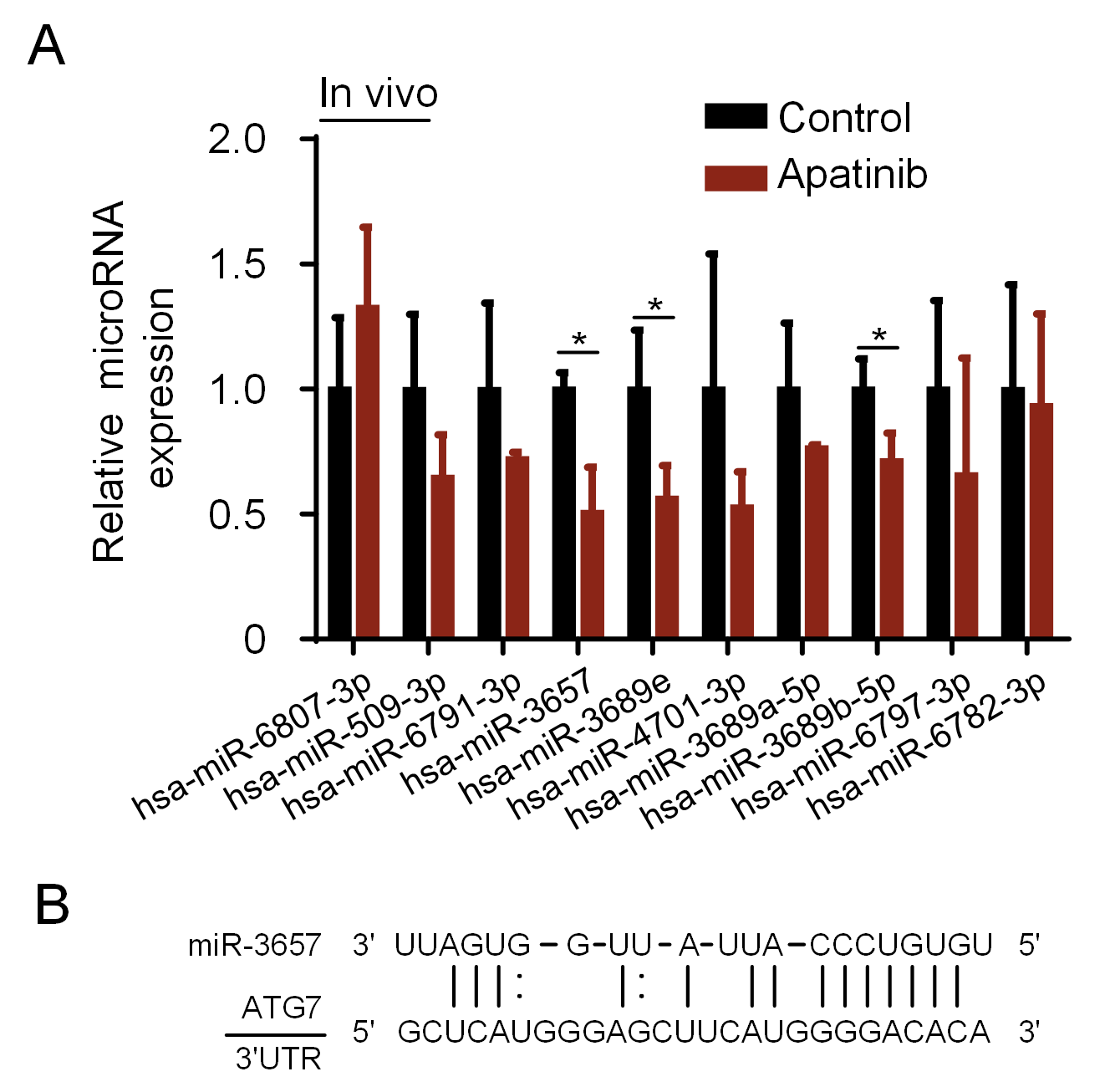


**Figure S3.** Supporting information for Figure 4. Supplementary data for miRNA-seq and prediction of binding sequences for *miR-3657*. (**A**) A qRT-PCR assay was conducted to confirm the expression of the ten downregulated miRNAs identified by miRNA-seq. *, *P* < 0.05. (**B**) The predicted binding sequences for *miR-3657* within the human *ATG7* 3’UTR identified by miRanda.


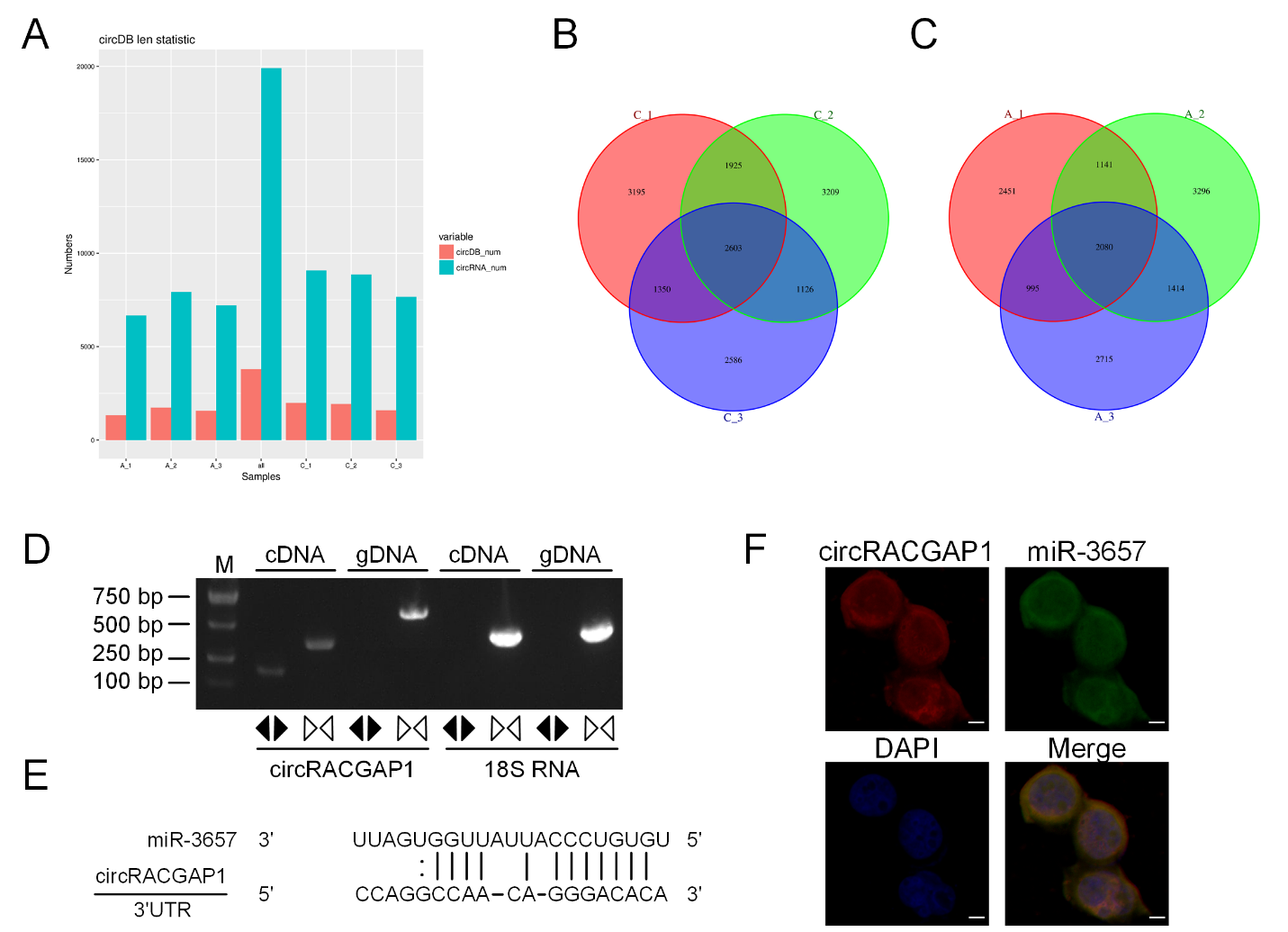


**Figure S4.** Supporting information for Figure 5. Supplementary data for circRNA-seq and prediction of binding sequences for *circRACGAP1*. (**A**) The circRNA candidates identified by circRNA-seq and those that have been found in circBase (circDB) are shown for each sequencing sample. (**B and C**) The Venn diagrams show the relationship between the circRNAs from sequencing samples in the control group (C_1, C_2, and C_3) and apatinib group (A_1, A_2, and A_3). (**D**) Divergent primers amplify *circRACGAP1* from cDNA but not genomic DNA (gDNA). 18S RNA as control. (**E**) The predicted binding sequences for *miR-3657* within the human *circRACGAP1* 3’UTR identified by miRanda. (F) Immunofluorescence imaging of *circRACGAP1* (red), *miR-3657* (green), nucleus labeled as DAPI (blue), the co-localization of the three signals (merge). Bar scale, 5 μm.


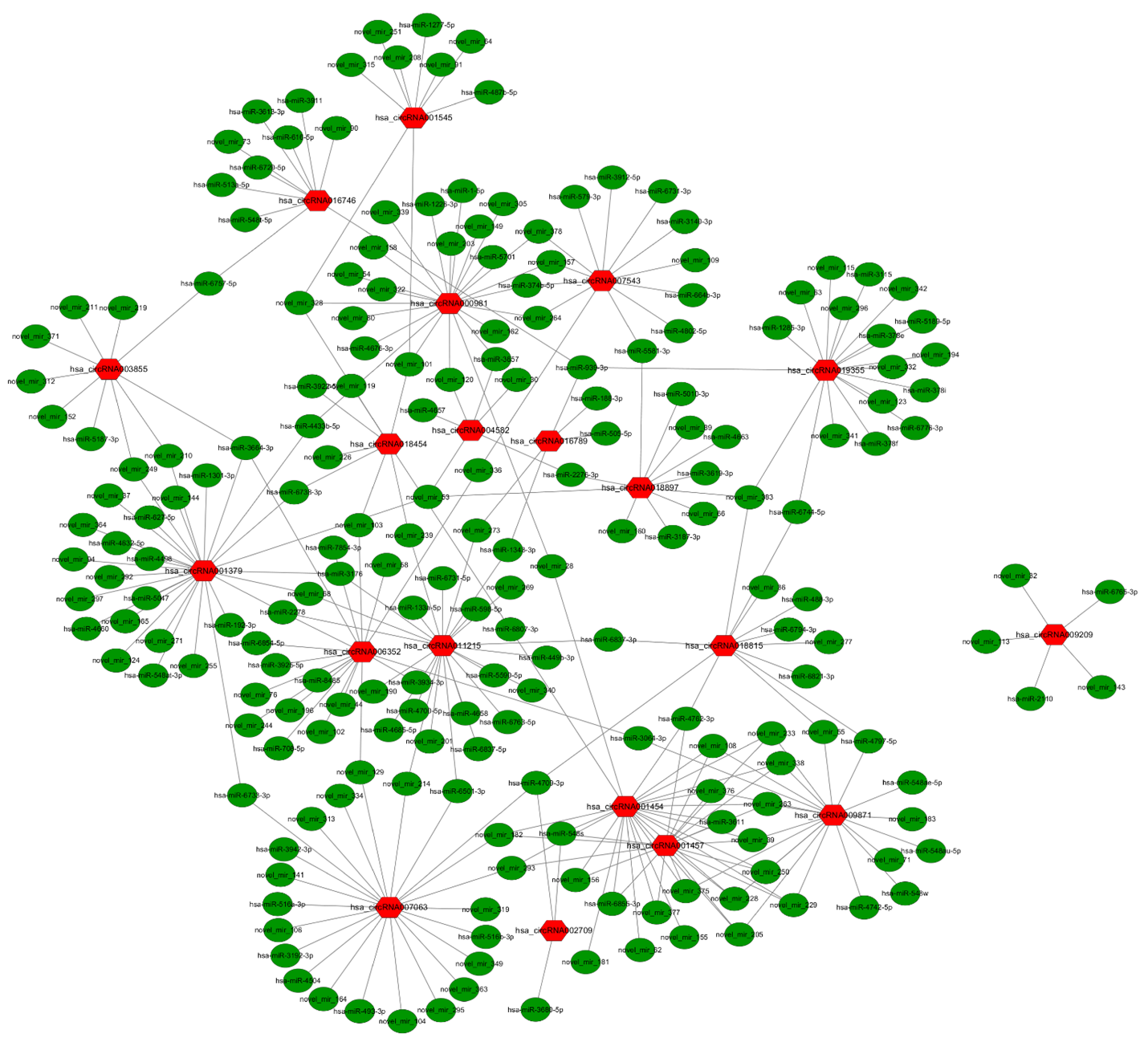


**Figure S5.** Supporting information for Figure 5. The circRNA-miRNA network for the twenty most differentially upregulated circRNAs. In the network, miRNAs (fold change ≤ -2) that possibly target the twenty circRNAs were collected. The circRNA/miRNA interaction was predicted using miRanda and TargetScan.


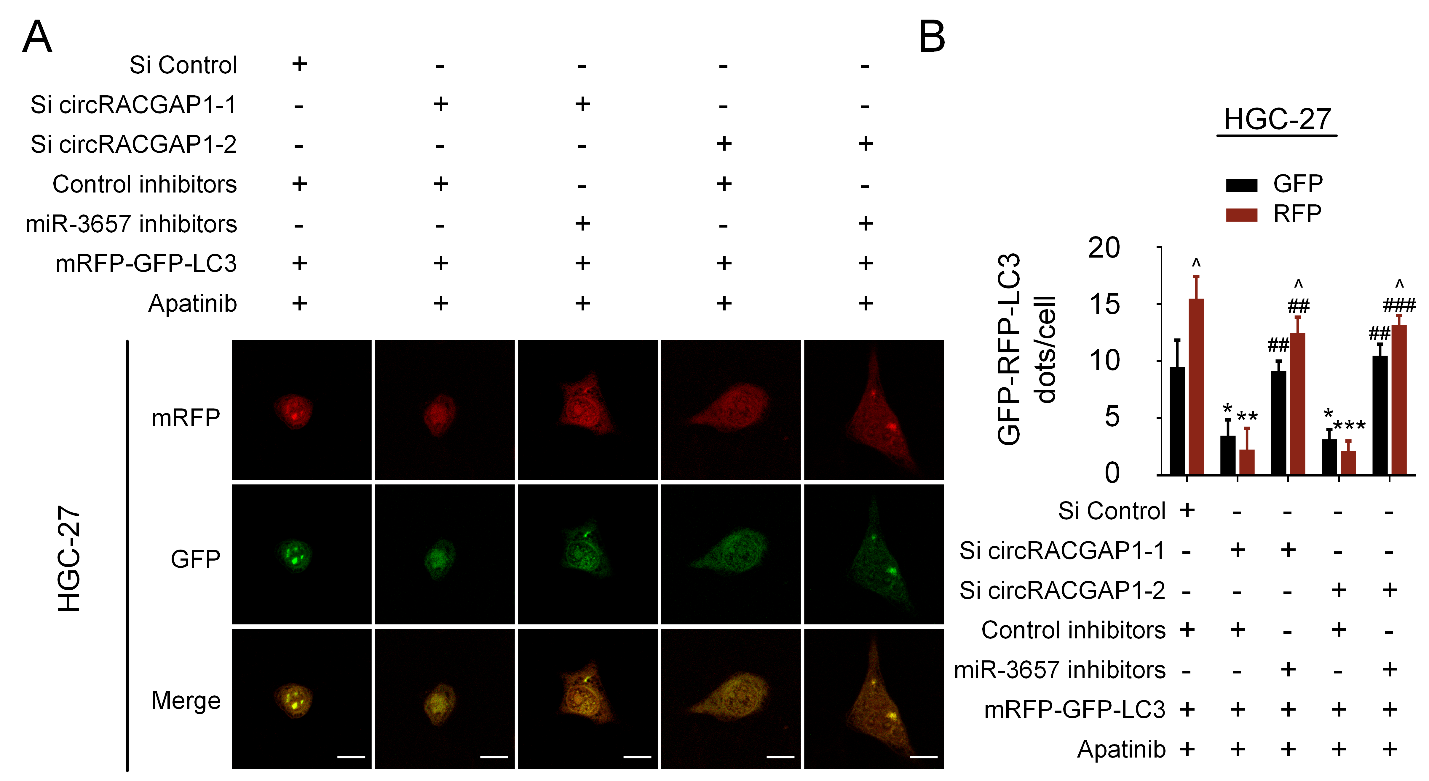


**Figure S6.** Supporting information for Figure 6. (**A**) HGC-27 cells were cotransfected with *circRACGAP1* siRNA or control siRNA, *miR-3657* inhibitors or control inhibitors, and mRFP-GFP-LC3 plasmid. After 24 h, the cells were incubated with 40 µM apatinib for 24 h and analyzed by confocal microscopy. Bar scale, 10 μm. (**B**) The numbers of GFP^+^ or RFP^+^ dots per cell are presented as the means ± SD of three independent experiments. *, *P* < 0.05; **, *P* < 0.01; ***, *P* < 0.001 compared with the group transfected with si control. ##, *P* < 0.01; ###, *P* < 0.001 compared with the group cotransfected with si *circRACGAP1*-1 or si *circRACGAP1*-2 and control inhibitors. ^, *P* < 0.05 compared with the corresponding GFP group.


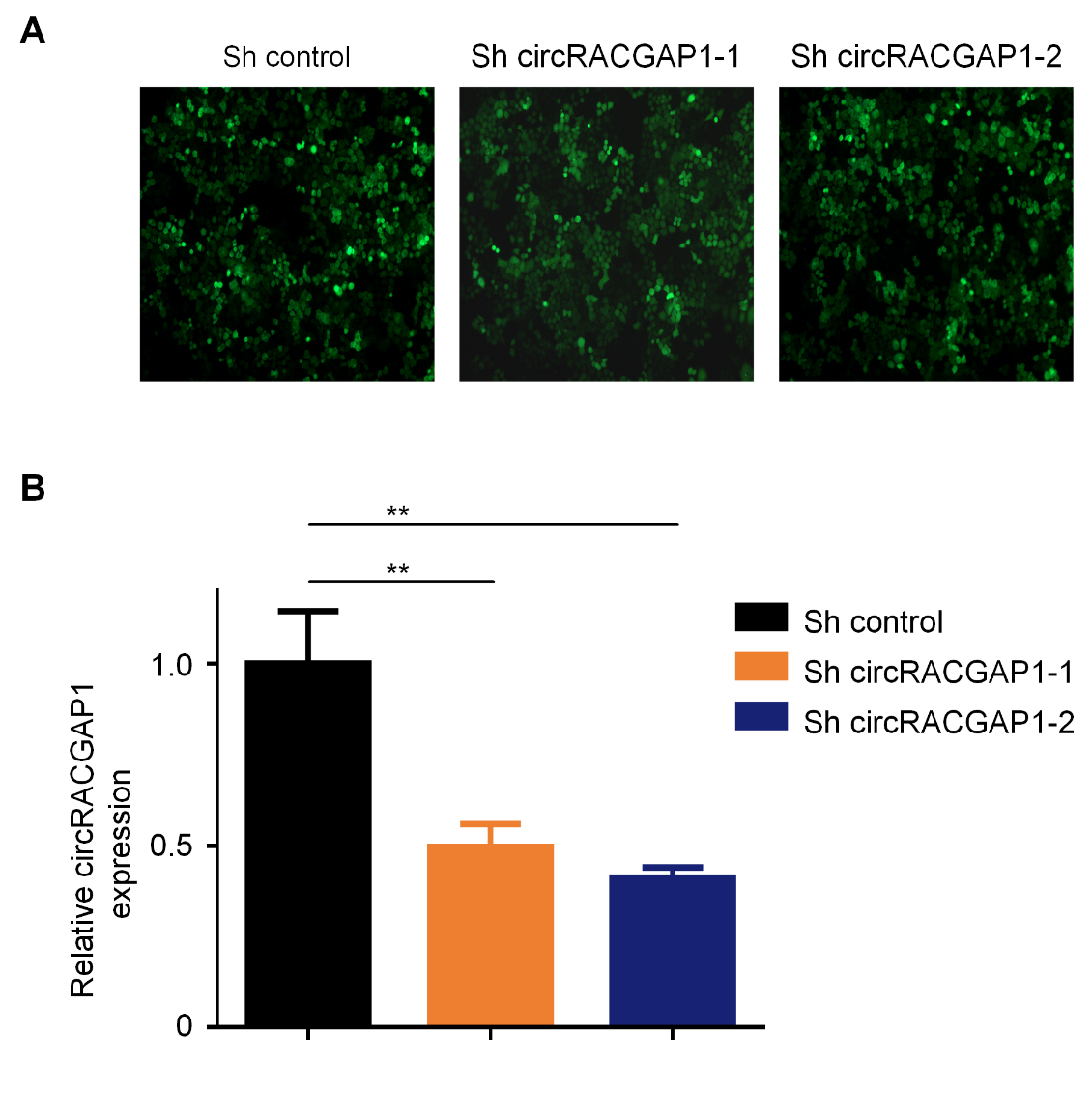


**Figure S7.** Supporting information for Figure 7. (**A**) BGC-823 cells were stably transfected with *circRACGAP1* shRNA or control shRNA lentivirus. The confocal images were acquired to visualize the transfection of GFP-positive BGC-823 cells. (**B**) A qRT-PCR assay was conducted to examine the expression of *circRACGAP1* after transfection with *circRACGAP1* shRNA lentivirus. **, *P* < 0.01.


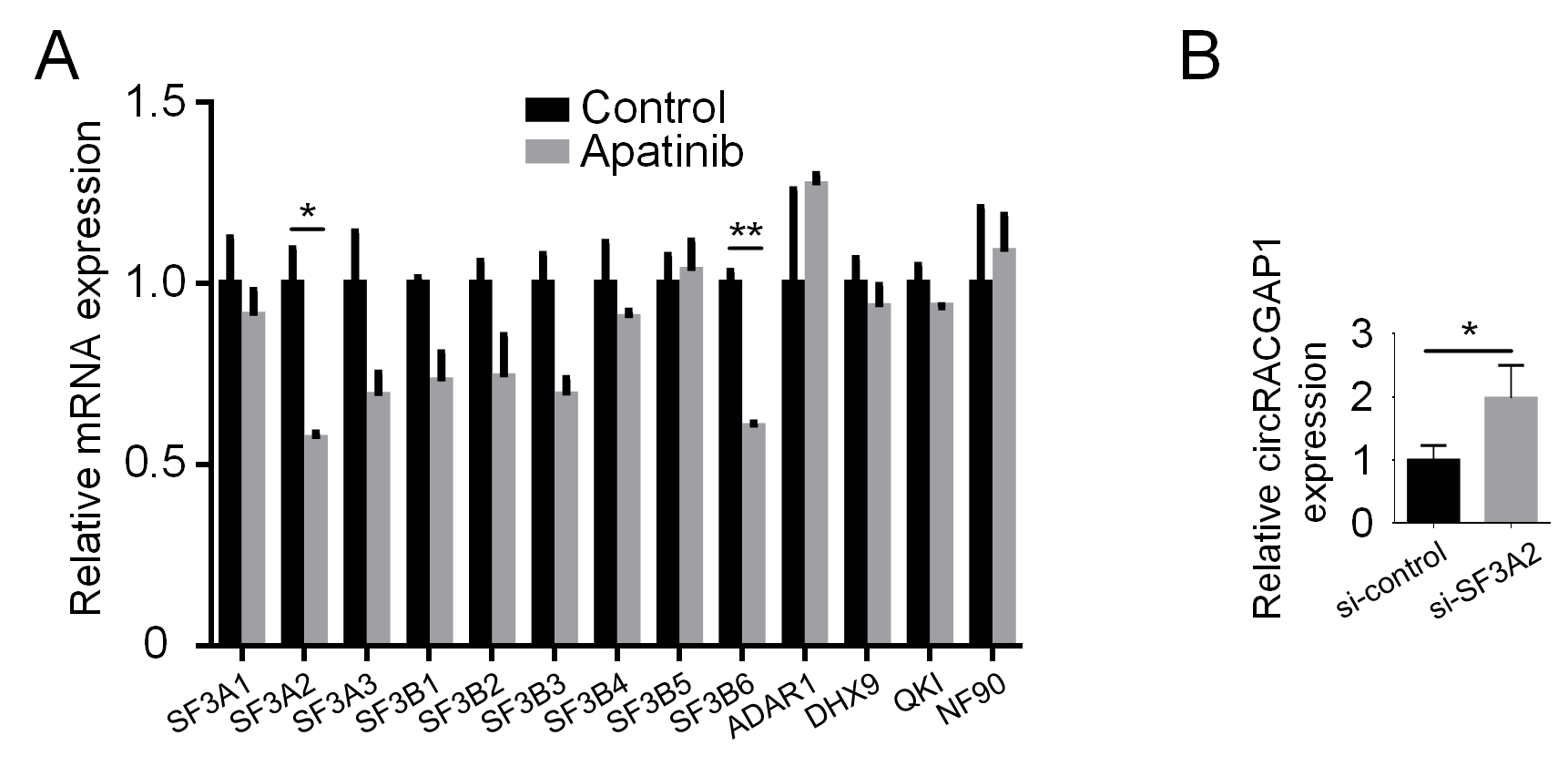


**Figure S8.** Apatinib decreased SF3A2 expression. (A) A qRT-PCR was performed in *vivo* to detect the change of the mRNA levels after apatinib treatment. (B) qRT-PCR was used in BGC-823 cells transfected with SF3A2 siRNA to detect the expression of *circRACGAP1*. *, *P* < 0.05; **, *P* < 0.01.

**Supplementary Table**

**Table S1. The primer sequences for qRT-PCR and the sequences of siRNAs.**

| Primer names | Sequences (5’-3’) |
| --- | --- |
| GAPDH-F | GGAGCGAGATCCCTCCAAAAT |
| GAPDH-R | GGCTGTTGTCATACTTCTCATGG |
| ATG7-F | CAGTTTGCCCCTTTTAGTAGTGC |
| ATG7-R | CCAGCCGATACTCGTTCAGC |
| ULK1-F | GGCAAGTTCGAGTTCTCCCG |
| ULK1-R | CGACCTCCAAATCGTGCTTCT |
| ATG2B-F | GGACGGTTAATTGGTAGGTTGG |
| ATG2B-R | CTGCATGGGTCGATTTTTCCT |
| ATG3-F | GACCCCGGTCCTCAAGGAA |
| ATG3-R | TGTAGCCCATTGCCATGTTGG |
| ATG4B-F | ATGGACGCAGCTACTCTGAC |
| ATG4B-R | TTTTCTACCCAGTATCCAAACGG |
| ATG5-F | AAAGATGTGCTTCGAGATGTGT |
| ATG5-R | CACTTTGTCAGTTACCAACGTCA |
| ATG8-F | ACTCGCTGGAACACAGATGC |
| ATG8-R | TCTGAGAGCCTGAGACCTTTT |
| ATG10-F | AGACCATCAAAGGACTGTTCTGA |
| ATG10-R | GGGTAGATGCTCCTAGATGTGAC |
| ATG12-F | CTGCTGGCGACACCAAGAAA |
| ATG12-R | CGTGTTCGCTCTACTGCCC |
| ATG13-F | TTGCTATAACTAGGGTGACACCA |
| ATG13-R | CCCAACACGAACTGTCTGGA |
| ATG14-F | GCGCCAAATGCGTTCAGAG |
| ATG14-R | AGTCGGCTTAACCTTTCCTTCT |
| ATG16L1-F | AACGCTGTGCAGTTCAGTCC |
| ATG16L1-R | AGCTGCTAAGAGGTAAGATCCA |
| circRACGAP1-F | TCAAGCGTCAACCCAAGGTG |
| Continued |  |
| circRACGAP1-R | ACATGGCAGCTATGCTGTTGT |
| hsa_circRNA007543-F | CAAGTTTTGCAGCACTGTGGC |
| hsa_circRNA007543-R | CACTGGGTTGCAGGAATATGCT |
| hsa_circRNA018454-F | TATGGTGGCAAAGCTGCTGG |
| hsa_circRNA018454-R | CGTTTGTAGCGAGGACGCAA |
| hsa_circRNA003855-F | AGGTGGATACCACACCAACAGT |
| hsa_circRNA003855-R | ACATTGCCTCCAACGCTGAT |
| hsa_circRNA002709-F | CTCGGCAGCATCATGGCTATT |
| hsa_circRNA002709-R | ACAGAGGGTCCTGCTAAGCG |
| hsa_circRNA016746-F | TCCCACAGTATCTCTATCCAGCCT |
| hsa_circRNA016746-R | CGCCCAGCCCTGTAACATCT |
| hsa_circRNA000981-F | TCGAAGACCCATTGGTAAGATGACA |
| hsa_circRNA000981-R | ACACCTTGTCCACCACAGCTC |
| hsa_circRNA018815-F | AGCTGGATGCTACTGGGATGT |
| hsa_circRNA018815-R | AGTCTCATAGCCACCAAACATCAT |
| hsa_circRNA011215-F | ACACAGCAGGACTGAGTGAAG |
| hsa_circRNA011215-R | TCTGGGGAGATATACAGACGGC |
| hsa_circRNA009209-F | CGGAGTTCCGGTACAAGCTC |
| hsa_circRNA009209-R | CTGTCGTGCAGCCAGAAGAC |
| hsa_circRNA018897-F | TGGCCAATCCACAAATGCAGC |
| hsa_circRNA018897-R | TCATGAATTCCATGCTGACTCAAGG |
| hsa_circRNA006352-F | AGGTAGAGATTGTTGGCATGTTGC |
| hsa_circRNA006352-R | TGATGTCCACGGAAGGGTCC |
| hsa_circRNA001545-F | AGGGTAGGGCTCTACTTCACTG |
| hsa_circRNA001545-R | CATCCACATTTTTCGATTTGATGGC |
| hsa_circRNA009871-F | CGAGTCTCAAAGGTGTTCCTGG |
| hsa_circRNA009871-R | GCCACAAGGCGGTTGTTGAT |
| hsa_circRNA007063-F | ACTGAGATTCCACTCCCCTCC |
| hsa_circRNA007063-R | AGGTCTCTTGTGCTCTGGAAGA |
| Continued |  |
| hsa_circRNA001379-F | GGGAGTCACACAGCTCCCTA |
| hsa_circRNA001379-R | TTGCAGCTCATCAGTGTCTGTG |
| 18S RNA-F | TGAGAAACGGCTACCACATCC |
| 18S RNA-R | GGCCTGCTTTGAACACTCTAATT |
| Divergent 18S RNA-F | TTAATTCCGATAACGAACGAGAC |
| Divergent 18S RNA-R | AGAGGAGCGAGCGACCAA |
| Convergent circRACGAP1-F | AGCATAGCTGCCATGTACC |
| Convergent circRACGAP1-R | TGTTGAAAAGGCATTTGAGT |
| Divergent circRACGAP1-F | TGATGGTGGAGCAAGAG |
| Divergent circRACGAP1-R | TTGGTACATGGCAGCTAT |
| SF3A1-F | GAGGATTCTGCACCTTCTAAGC |
| SF3A1-R | AGTAGGCATGGTAAGGGTCATT |
| SF3A2-F | CCACGTCACCGCTTCATGTCTG |
| SF3A2-R | GAACTTGCCCTCCGCCTTGTC |
| SF3A3-F | GTCATGGCTAAAGAGATGCTCAC |
| SF3A3-R | TCCTCCTTTCGTAATCCATCCTT |
| SF3B1-F | GTGGGCCTCGATTCTACAGG |
| SF3B1-R | GATGTCACGTATCCAGCAAATCT |
| SF3B2-F | GGCCCCACTCCTACAGTTTTG |
| SF3B2-R | GGACGAGCGAGCATCTGTTT |
| SF3B3-F | GCCATTCATGGAAACTTTTCTGG |
| SF3B3-R | GTGAGTAGGGTATGTACTTTGCC |
| SF3B4-F | CTCCGAGCGGAATCAGGATG |
| SF3B4-R | GGCATGTGGGTGTTGACTACT |
| SF3B5-F | ACTGACCGCTACACCATCCAT |
| SF3B5-R | GTAGTTGAGAAGGTCGAAGTGG |
| SF3B6-F | GCCAAGAGGGCGAACATTC |
| SF3B6-R | TCCTCATAGACCACATAAGCTGT |
| ADAR1-F | CTGAGACCAAAAGAAACGCAGA |
| Continued |  |
| ADAR1-R | GCCATTGTAATGAACAGGTGGTT |
| DHX9-F | CGAACCATCTCAGCGACAAAA |
| DHX9-R | TGAGGTCCATGCTTATTTGCTC |
| QKI-F | AAGCCCACCCCAGATTACCT |
| QKI-R | ACTCTGCTAATTTCTTCGTCCAG |
| NF90-F | AGCATTCTTCCGTTTATCCAACA |
| NF90-R | GCTCGTCTATCCAGTCGGAC |
| SiRNAs | Sequences (5’-3’) |
| Si ATG7-1 | GGAACACTGTATAACACCA |
| Si ATG7-2 | CCAAAGTTCTTGATCAATA |
| Si circRACGAP1-1 | CACCAGATATTAAAGAAAT |
| Si circRACGAP1-2 | CCAGATATTAAAGAAATCA |

Abbreviations: F, Forward; R, Reverse.
